# The Contribution of HCN Channelopathies in Different Epileptic Syndromes, Mechanisms, Modulators, and Potential Treatment Targets: A Systematic Review

**DOI:** 10.3389/fnmol.2022.807202

**Published:** 2022-05-19

**Authors:** Miriam Kessi, Jing Peng, Haolin Duan, Hailan He, Baiyu Chen, Juan Xiong, Ying Wang, Lifen Yang, Guoli Wang, Karlmax Kiprotich, Olumuyiwa A. Bamgbade, Fang He, Fei Yin

**Affiliations:** ^1^Department of Pediatrics, Xiangya Hospital, Central South University, Changsha, China; ^2^Hunan Intellectual and Developmental Disabilities Research Center, Changsha, China; ^3^Department of Pediatrics, Kilimanjaro Christian Medical University College, Moshi, Tanzania; ^4^Department of Epidemiology and Medical Statistics, School of Public Health, Moi University, Eldoret, Kenya; ^5^Department of Anesthesiology and Pharmacology, University of British Columbia, Vancouver, BC, Canada

**Keywords:** HCN channelopathies, epilepsy, acquired channelopathy, neuro-inflammation, SUDEP

## Abstract

**Background:**

Hyperpolarization-activated cyclic nucleotide-gated (HCN) current reduces dendritic summation, suppresses dendritic calcium spikes, and enables inhibitory GABA-mediated postsynaptic potentials, thereby suppressing epilepsy. However, it is unclear whether increased HCN current can produce epilepsy. We hypothesized that gain-of-function (GOF) and loss-of-function (LOF) variants of HCN channel genes may cause epilepsy.

**Objectives:**

This systematic review aims to summarize the role of HCN channelopathies in epilepsy, update genetic findings in patients, create genotype–phenotype correlations, and discuss animal models, GOF and LOF mechanisms, and potential treatment targets.

**Methods:**

The review was conducted in accordance with the Preferred Reporting Items for Systematic Reviews and Meta-Analyses statement, for all years until August 2021.

**Results:**

We identified pathogenic variants of *HCN1* (*n* = 24), *HCN2* (*n* = 8), *HCN3* (*n* = 2), and *HCN4* (*n* = 6) that were associated with epilepsy in 74 cases (43 *HCN1*, 20 *HCN2*, 2 *HCN3*, and 9 *HCN4*). Epilepsy was associated with GOF and LOF variants, and the mechanisms were indeterminate. Less than half of the cases became seizure-free and some developed drug-resistant epilepsy. Of the 74 cases, 12 (16.2%) died, comprising *HCN1* (*n* = 4), *HCN2* (*n* = 2), *HCN3* (*n* = 2), and *HCN4* (*n* = 4). Of the deceased cases, 10 (83%) had a sudden unexpected death in epilepsy (SUDEP) and 2 (16.7%) due to cardiopulmonary failure. SUDEP affected more adults (*n* = 10) than children (*n* = 2). *HCN1* variants p.M234R, p.C329S, p.V414M, p.M153I, and p.M305L, as well as *HCN2* variants p.S632W and delPPP (p.719–721), were associated with different phenotypes. *HCN1* p.L157V and *HCN4* p.R550C were associated with genetic generalized epilepsy. There are several HCN animal models, pharmacological targets, and modulators, but precise drugs have not been developed. Currently, there are no HCN channel openers.

**Conclusion:**

We recommend clinicians to include *HCN* genes in epilepsy gene panels. Researchers should explore the possible underlying mechanisms for GOF and LOF variants by identifying the specific neuronal subtypes and neuroanatomical locations of each identified pathogenic variant. Researchers should identify specific HCN channel openers and blockers with high binding affinity. Such information will give clarity to the involvement of HCN channelopathies in epilepsy and provide the opportunity to develop targeted treatments.

## Introduction

Epilepsy is a common neurological disorder with a lifetime prevalence of 7.60 per 1,000 persons (Fiest et al., 2017). The types with the highest prevalence are epilepsy of unknown cause and generalized seizures (Fiest et al., 2017). It is estimated that there are 50 million people with epilepsy globally, of whom 125,000 die annually and 13 million develop disabilities (Singh and Sander, 2020). Sudden unexpected death in epilepsy (SUDEP) is a cause of death with an estimated incidence of 1 case per 10,000 patient-years for newly diagnosed epilepsy, 1–2 cases per 1,000 patient-years for chronic epilepsy, and 2–10 cases per 1,000 patient-years for drug-resistant epilepsy (Shankar et al., 2017). The causes of SUDEP are not clear, but most cases present with postictal cardiorespiratory dysfunction, and the commonest risk factor is a previous history of generalized tonic–clonic seizures (Shankar et al., 2017). With advanced genomic sequencing methods, new channel genes that are related to epilepsy have been discovered, including calcium (Noebels, 2012; Kessi et al., 2021), potassium (Brenner and Wilcox, 2012; Kessi et al., 2020a), and HCN (hyperpolarization-activated, cyclic nucleotide-gated) channels.

Hyperpolarization-activated cyclic nucleotide-gated channels are the types of non-selective cation channels which are mainly found in the neurons and heart. They can alter the intrinsic and synaptic excitability of principal neurons and GABAergic interneurons (Albertson et al., 2013; Zhao et al., 2016; Bohannon and Hablitz, 2018). Hippocampal excitatory neurons and hippocampal somatostatin-expressing interneurons express HCN channels commonly in the soma and dendrites, whereas parvalbumin-positive interneurons (GABAergic interneurons) express HCN channels solely in axons and nerve terminals (Roth and Hu, 2020; Speigel et al., 2022). HCN channels belong to a six transmembrane-ion channel family and are activated by membrane hyperpolarization (Benarroch, 2013). They conduct mixed cation, sodium, and potassium ion currents. They may form channels in a homomeric or heteromeric manner (Benarroch, 2013). HCN channels have a highly efficient cyclic nucleotide-binding domain (CNBD) at the C terminus (intrinsic regulatory site), which confers an isoform-specific sensitivity to cyclic AMP (cAMP) (Biel et al., 2009; Rivolta et al., 2020). HCN channels are enhanced by the direct binding of cAMP (Herrmann et al., 2007). HCN2 and HCN4 isoforms are most sensitive to cAMP, followed by HCN1, but HCN3 is not sensitive (Santoro and Shah, 2020). CNBD has an inhibitory effect on HCN channel gating (Tsay et al., 2007). Minor differences in the energies of the closed and open states of HCN channels result from different interactions between the voltage sensor and the pore; this explains why they are only activated during hyperpolarization (Ramentol et al., 2020). They produce currents that are termed as “If” in the heart and “Ih” in the brain (Benarroch, 2013). The currents are produced by 4 subtypes, namely, HCN1–4; each subtype is made up of four polymers consisting of six transmembrane domains, namely, S1-6, and intracellular amino and carboxyl termini (Sartiani et al., 2017). There is a pore-forming region between S5 and S6; S4 forms a voltage sensor (Sartiani et al., 2017). HCN channels have some intracellular auxiliary interacting proteins, including tetratricopeptide repeat-containing Rab8b-interacting protein (TRIP8b), His321, S4-S5 linker, pH sensitivity region, C-linker (subunit–subunit interactions), and Tyr476 (Src phosphorylation site) and extracellular auxiliary interacting proteins, including N-glycosylation site (Rivolta et al., 2020).

HCN channels, can regulate neuronal excitability. Therefore, any dysregulation of these channels can play a role in epileptogenesis. HCN channels are more expressed in the dendrites and are important for dendritic integration or regulation of synaptic currents, by changing the membrane resistance (Lewis et al., 2010; Noam et al., 2011). Ih-mediated depolarization inhibits calcium influx in T-type calcium channels, thereby interfering with synaptic release in the axon terminals of layer 3 entorhinal cortex neurons (Huang et al., 2011). Ih currents also exist in presynaptic membranes, but their role in the human brain is not clear (Noam et al., 2011). Dendritic Ih current also modulates the conduction of ions in other channels, including voltage-gated calcium channels (T-type and N-type) and rectifier M-type potassium channels (Tsay et al., 2007; George et al., 2009). It is known that Ih current can reduce dendritic summation, suppress dendritic calcium spikes, and enhance inhibitory GABAA-mediated postsynaptic potentials in pyramidal neurons (Noam et al., 2011), thereby preventing the occurrence of epilepsy. Despite the fact that loss-of-function (LOF) variants can eliminate dendritic attenuation in pyramidal neurons and contribute to hyperexcitability, it is generally known that epilepsies are more likely to result from a loss of the inhibitory component than from a gain of the excitatory component. It is unclear whether epilepsy can occur due to gain of the excitatory component, and if so, the specific neuroanatomical location of the gain-of-function (GOF) variants and distribution patterns (soma, dendrites, axons, or nerve terminals) are yet to be unveiled. We hypothesized that both GOF and LOF variants of HCN channel genes in different neurons can cause epilepsy, and that acquired HCN channelopathy can play a role in the pathogenesis of other epileptic syndromes.

This article provides a comprehensive review of the role of HCN channelopathies in different epileptic syndromes. It updates relevant information regarding human genetic changes, genotype–phenotype correlations, animal models, GOF or LOF mechanisms, and potential treatment targets. It highlights the implications of HCN channelopathies in other epilepsy syndromes, such as temporal lobe epilepsy, febrile seizures, Rett syndrome, absence seizures, malformation of cortical development, and febrile infection-related epilepsy syndrome (FIRES). The article discusses the role of HCN channelopathies in the occurrence of SUDEP.

## Methods

### Literature Search and Selection

The systematic review was conducted in accordance with the Preferred Reporting Items for Systematic Reviews and Meta-Analyses (PRISMA) statement (Moher et al., 2009). A thorough literature search was performed in PubMed and EMBASE, covering all the years until August 2021. A hand-search of the reference lists of published articles was also performed. Only the papers published in English were included. The search terms included the combination of the HCN channel and epilepsy or seizures or convulsions (Data Sheet 1). A librarian was consulted for the creation of search strategies. The two independent reviewers searched the articles to select the papers that meet the selection requirements.

The articles selected were cohort studies, case–control studies, cross-sectional studies, case series, and case reports. The review included published studies regarding epilepsy, HCN channel gene mutations, HCN channels related to auxiliary subunits, animal models, cell line model modulators, and treatments. The review also included articles that are related to HCN channelopathies with other epileptic syndromes, such as temporal lobe epilepsy, febrile seizures, Rett syndrome, absence seizures, and malformation of cortical development. The review excluded articles about epileptic cases that were associated with other types of channelopathies (sodium, potassium, calcium, and chloride) or other gene mutations. It also excluded abstracts, reviews, patents, book chapters, and conference papers.

### Data Extraction

The two independent reviewers scrutinized the article titles and abstracts and then read the full texts of the articles that met the inclusion criteria. The accuracy of the extracted information was guaranteed through team discussion and agreement. The major outcome measures of this review included the demographics of epileptic cases that are associated with HCN channelopathies (sex, age at seizure onset), initial seizure semiology, seizure types during the disease course, epileptic syndrome or phenotype, the presence or absence of status epilepticus, other clinical features or organ disorders, nucleotide or protein change, mode of inheritance, altered protein function (GOF or LOF), brain imaging results, electroencephalography findings, therapies used, prognosis, and the corresponding references. All identified HCN epilepsy-associated genes were further studied in OMIM, PubMed, and ClinVar databases to identify their function, expression, animal and cell model study outcomes, available treatments, pharmacological targets, and possible mechanisms of epilepsy.

### Data Analysis

The data were entered, processed, and analzsed using IBM^®^ SPSS^®^ Statistics 22 (IBM Corp, Armonk, NY). Data are summarized and presented as the mean age of onset, sex, seizure semiology, seizure outcome, disease course, therapies, and other outcome measures.

## Results

The initial literature search yielded 468 articles. Following the elimination of duplicates and articles that lacked full texts and/or were non-English, 199 remained eligible. All full texts were read and screened for eligibility. The articles that met all the inclusion criteria were 119, of which 14 were clinical studies and the remaining 105 involved animal studies, cell model studies, regulators, and pharmacology studies. The flowchart can be found in Supplementary Material.

### HCN Channelopathies Associated With Epilepsy and Their Functional Properties

**Figure 1:**
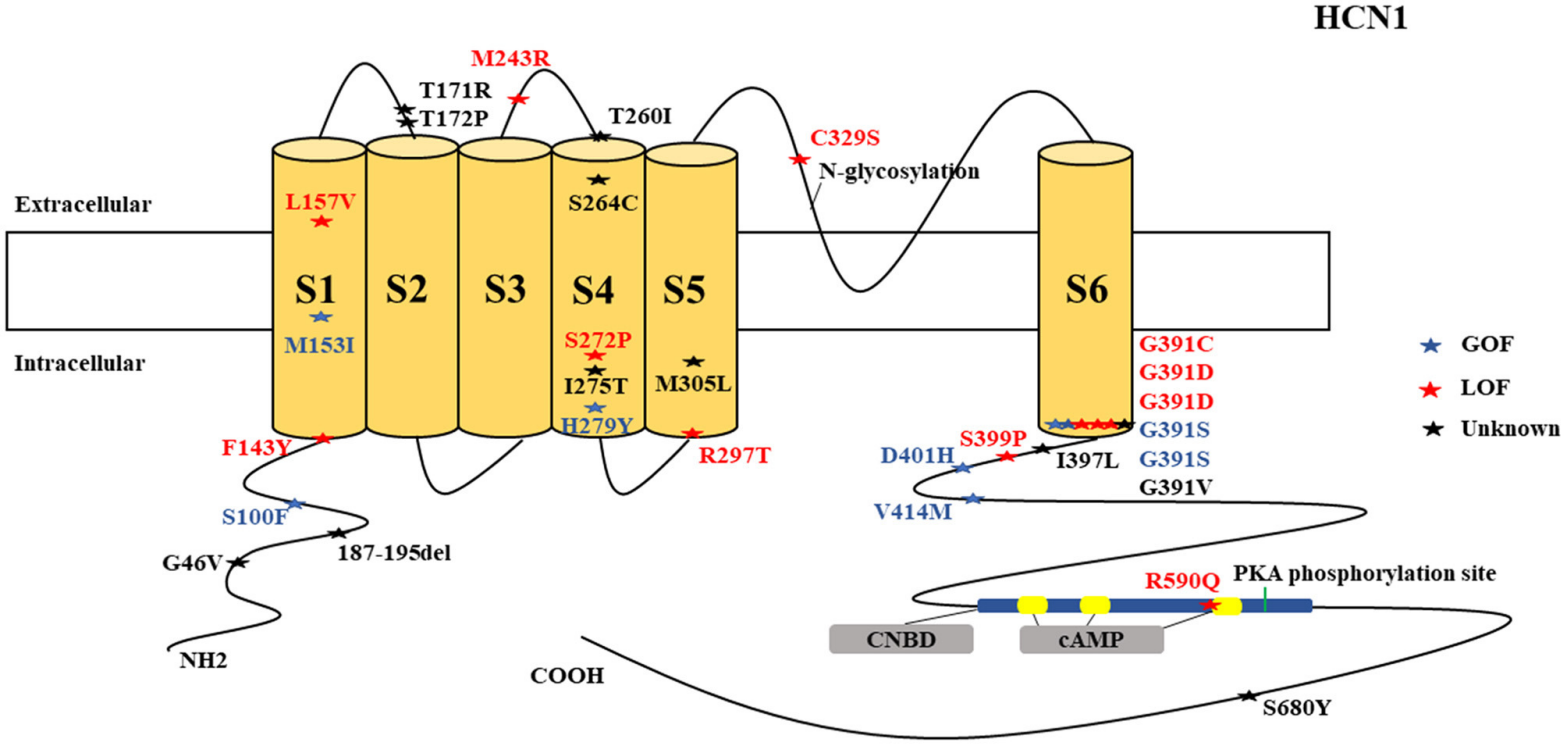
Schematic presentation of the HCN1 channel with 6 transmembrane domains (S1–S6), the locations of the pathogenic variants related to epilepsy and the altered protein functions. Most of mutations are located in S6, the intracellular linker between S6 and CNBD as well as in N-terminal. Notably, variants related to both epilepsy and SUDEP (p.G391D, p.G46V, and 187-195del) are located both in the N- and C-terminals and there is a hotspot in residue G391. Variants in blue correspond to gain-of-function (GOF) effects, variants in red correspond to loss-of-function (LOF) effects, and variants in black stand for the variants with unknown or clear effects.

**Figure 2:**
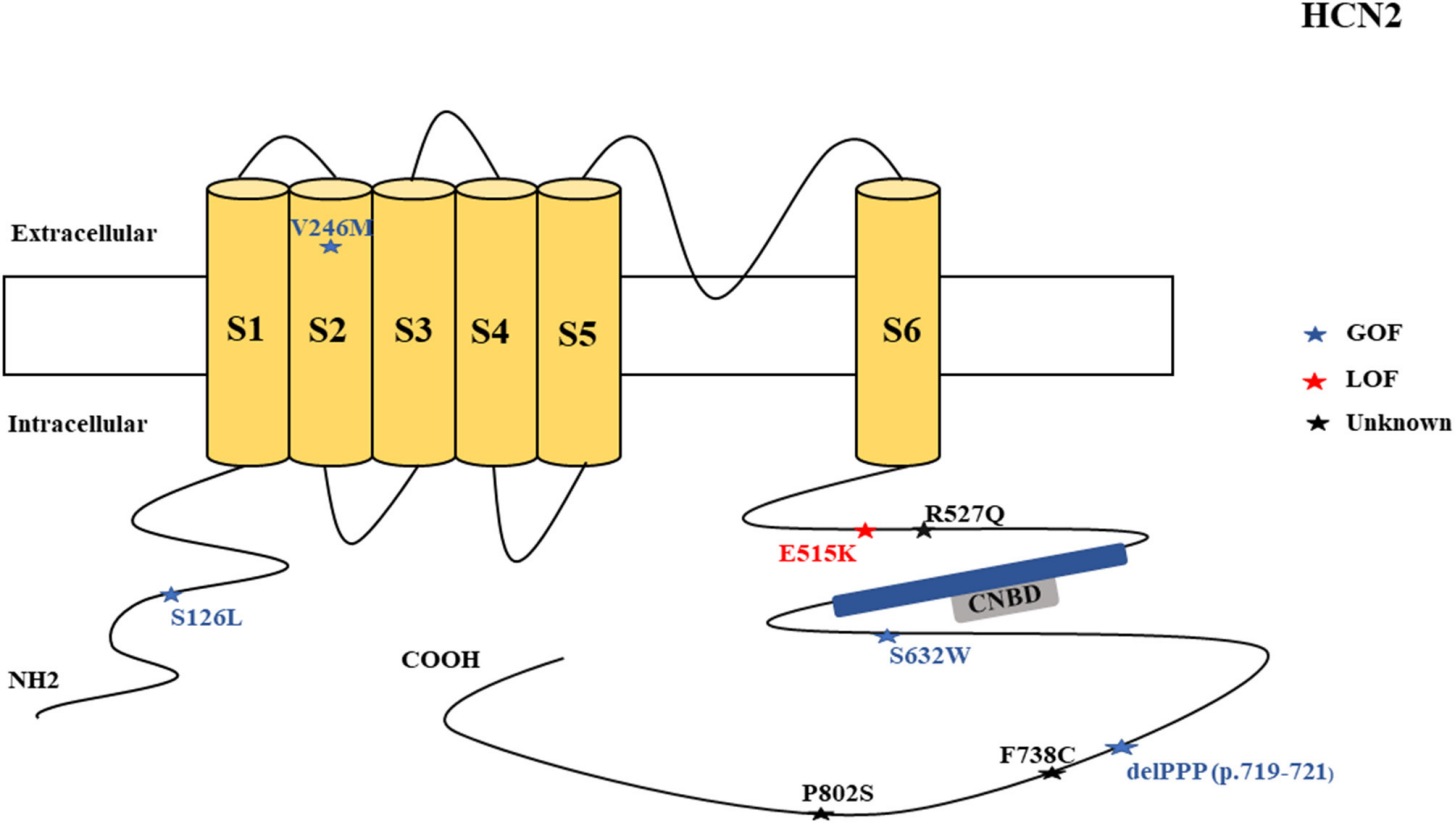
Schematic presentation of the HCN2 channel with 6 transmembrane domains (S1–S6), the locations of the pathogenic variants related to epilepsy, and the altered protein functions. Most of the mutations are located in the intracellular linker before and after the CNBD region. Notably, variants related to both epilepsy and SUDEP are located in C-terminal (p.F738C and p.P802S). Variants in blue correspond to gain-of-function (GOF) effects, variants in red correspond to loss-of- function (LOF) effects, and variants in black stand for the variants with unknown or clear effects.

**Figure 3:**
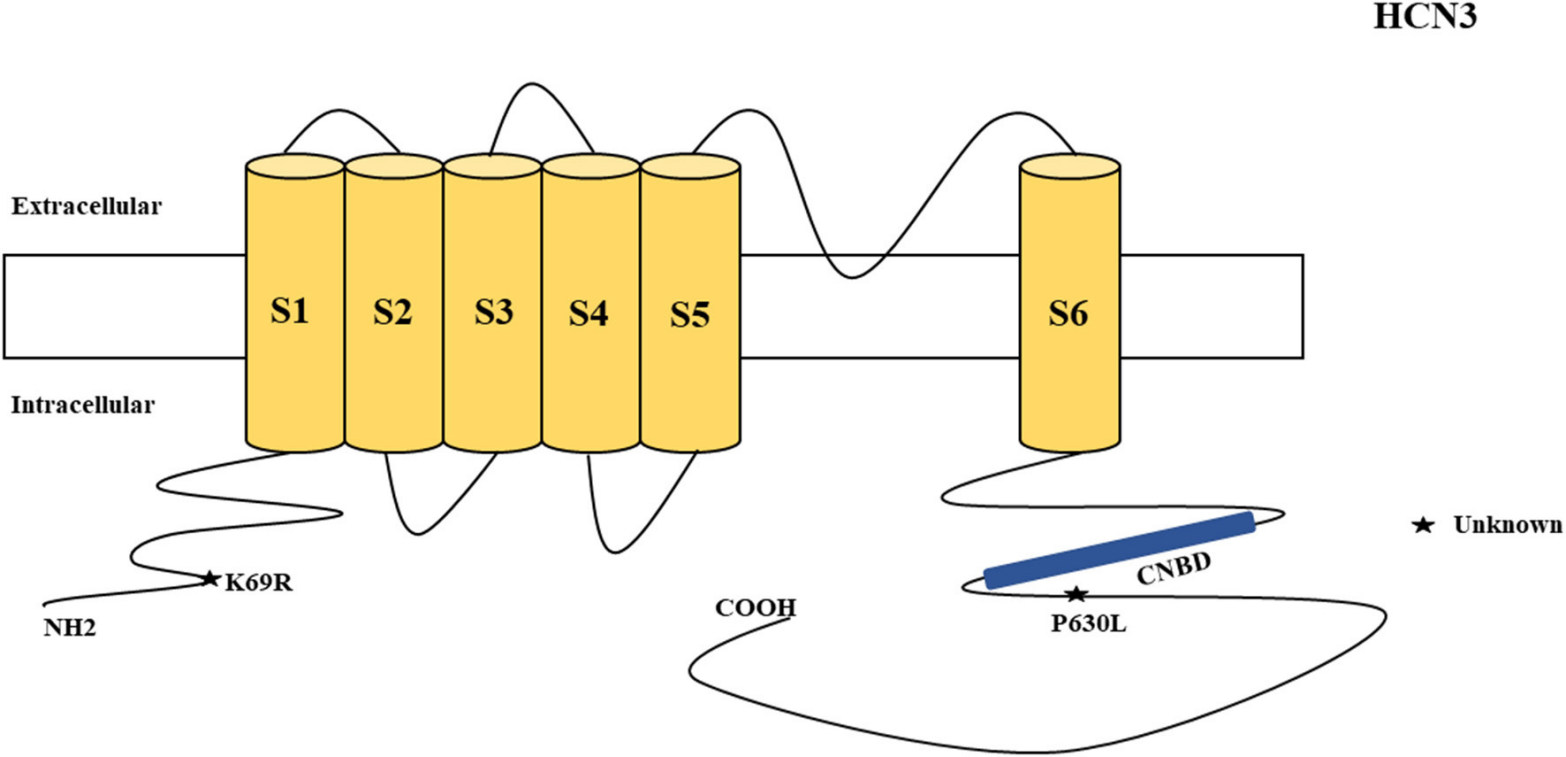
Schematic presentation of the HCN3 channel with 6 transmembrane domains (S1–S6) and the locations of the pathogenic variants related to epilepsy. Variants in black have unknown functional effects and both of them are related to both epilepsy and SUDEP.

**Figure 4:**
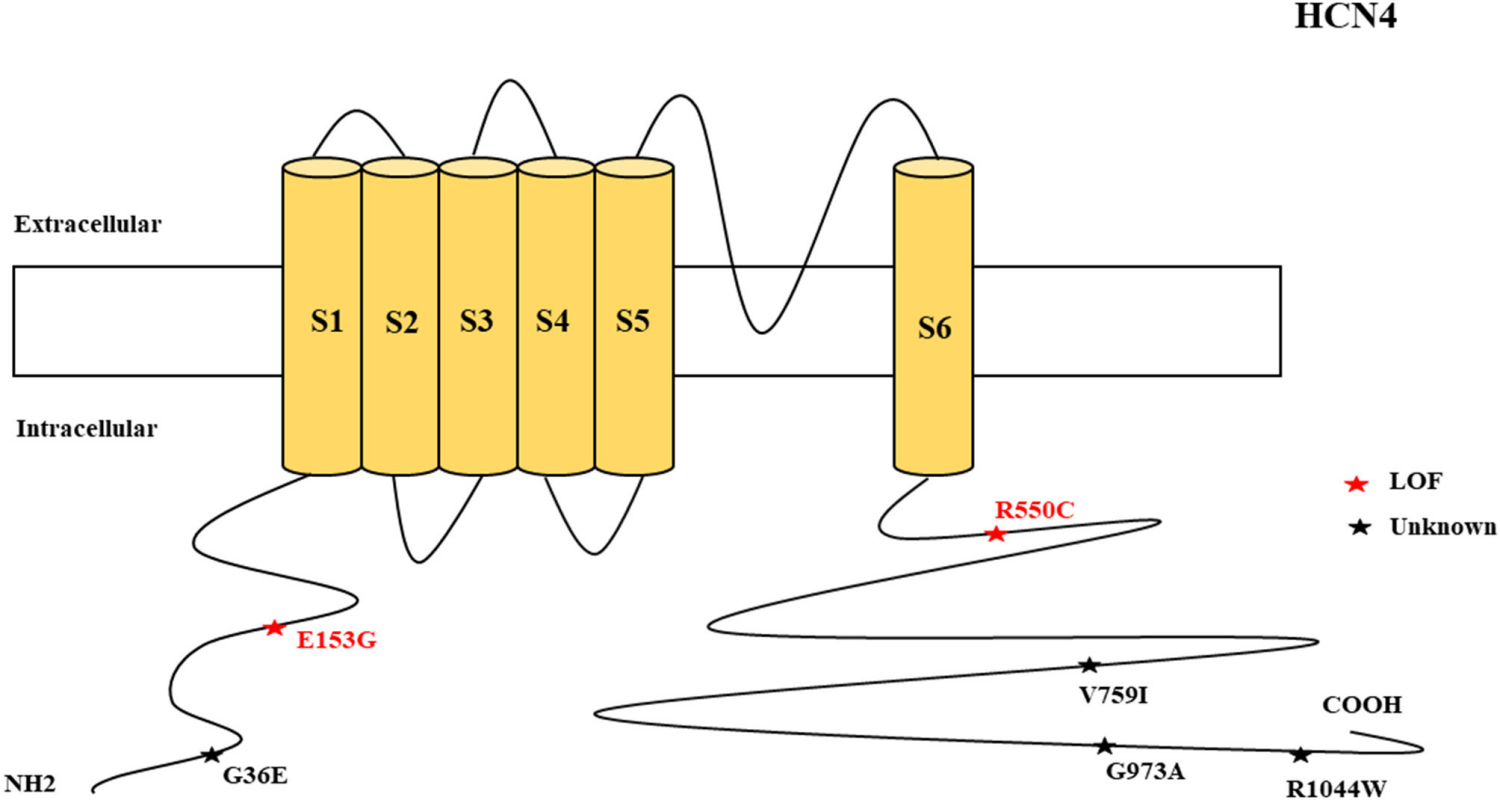
Schematic presentation of the HCN4 channel with 6 transmembrane domains (S1–S6), the locations of the pathogenic variants related to epilepsy, and the altered protein functions. Most of the mutations are located in C-terminal, including those related to both epilepsy and SUDEP (p.G36E, p.V759I, p.G973R, and p.R1044W). Variants in blue correspond to gain-of-function (GOF) effects, variants in red correspond to loss-of-function (LOF) effects, and variants in black stand for the variants with unknown or clear effects.

**Figure 5:**
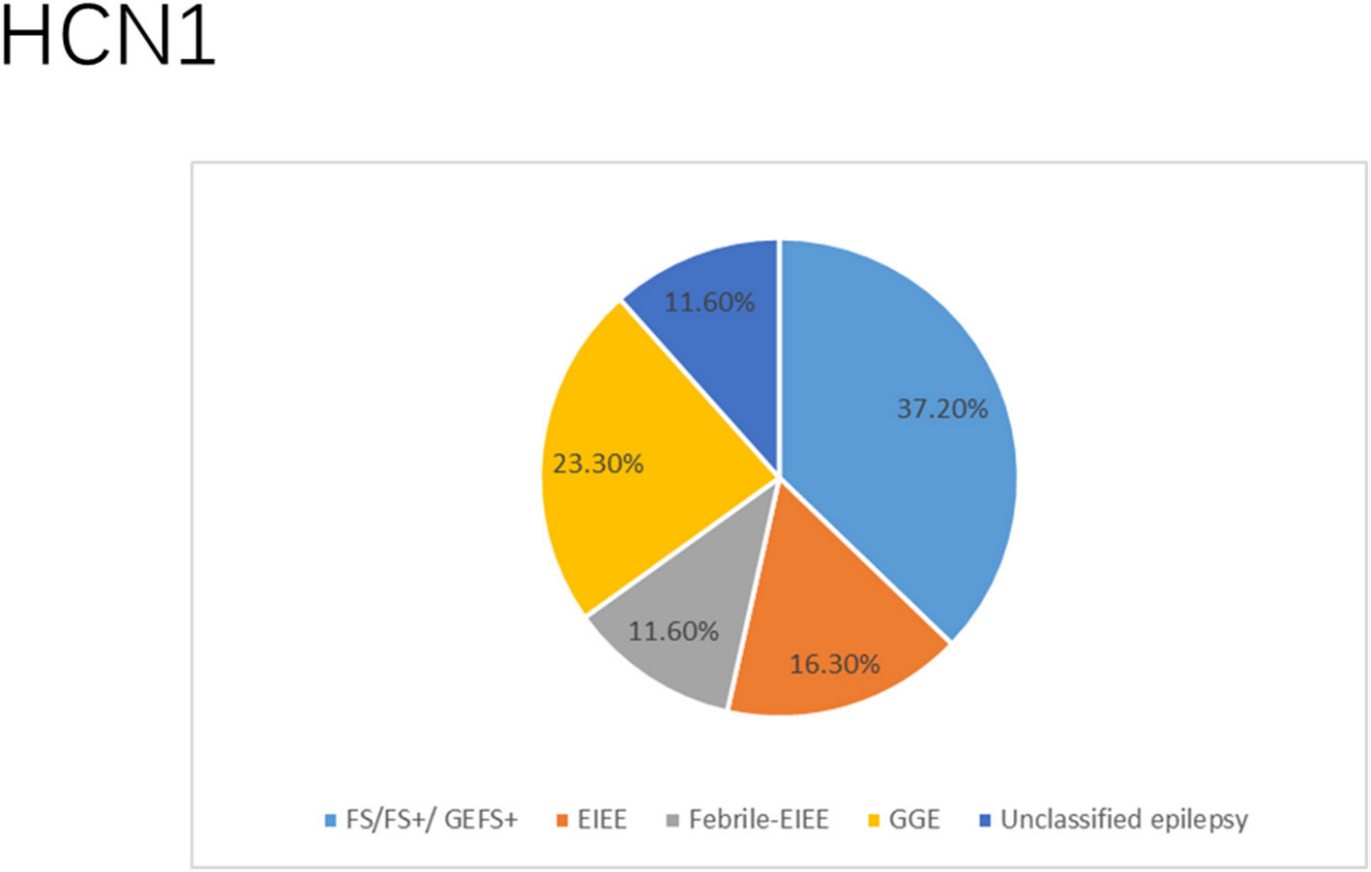
Clinical phenotypes related to *HCN1* variants. Most cases presented with febrile seizures (FS), or febrile seizure plus (FS+) or genetic generalized epilepsy with febrile seizure plus (GEFS+) followed by genetic or idiopathic generalized epilepsy (GGE), early infantile epileptic encephalopathy (EIEE), febrile EIEE, and few had unclassed epileptic syndromes (including those who died due to SUDEP and those reported to have unclassified epilepsy infantile).

**Figure 6:**
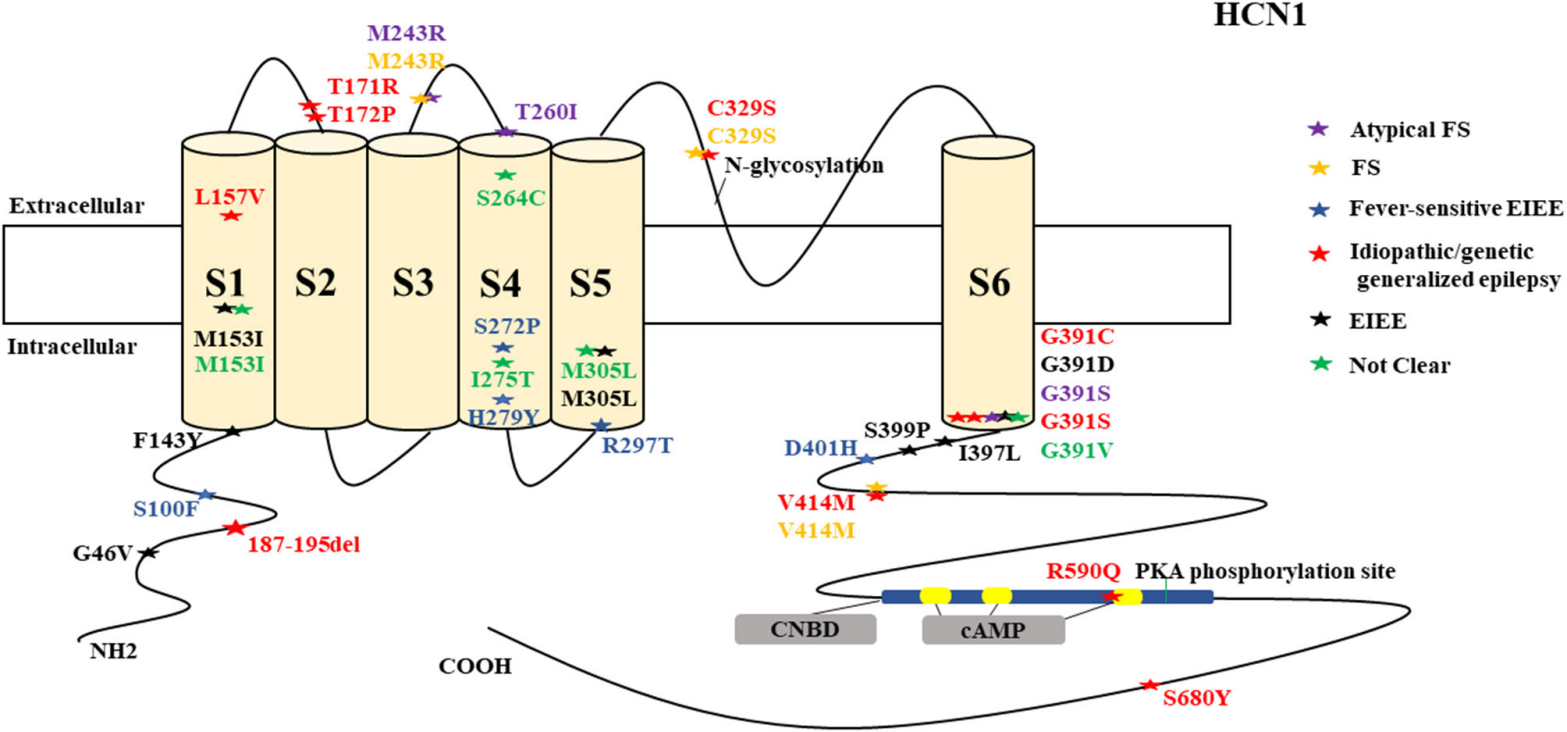
Schematic representation of *HCN1* variants related to different clinical epileptic phenotypes. Some of the variants are related to different epileptic syndromes: p.M234R is associated with both typical and atypical febrile seizures, p.C329S and p.V414M are each related to both febrile seizures and genetic or idiopathic generalized epilepsy, p.M153I and p.M305L are each related to both EIEE and unclassified epilepsy which occurs in infants. Atypical febrile seizures group includes cases with febrile seizure plus and genetic generalized epilepsy with febrile seizure plus. FS stands for febrile seizures and EIEE for early infantile epileptic encephalopathy.

**Figure 7:**
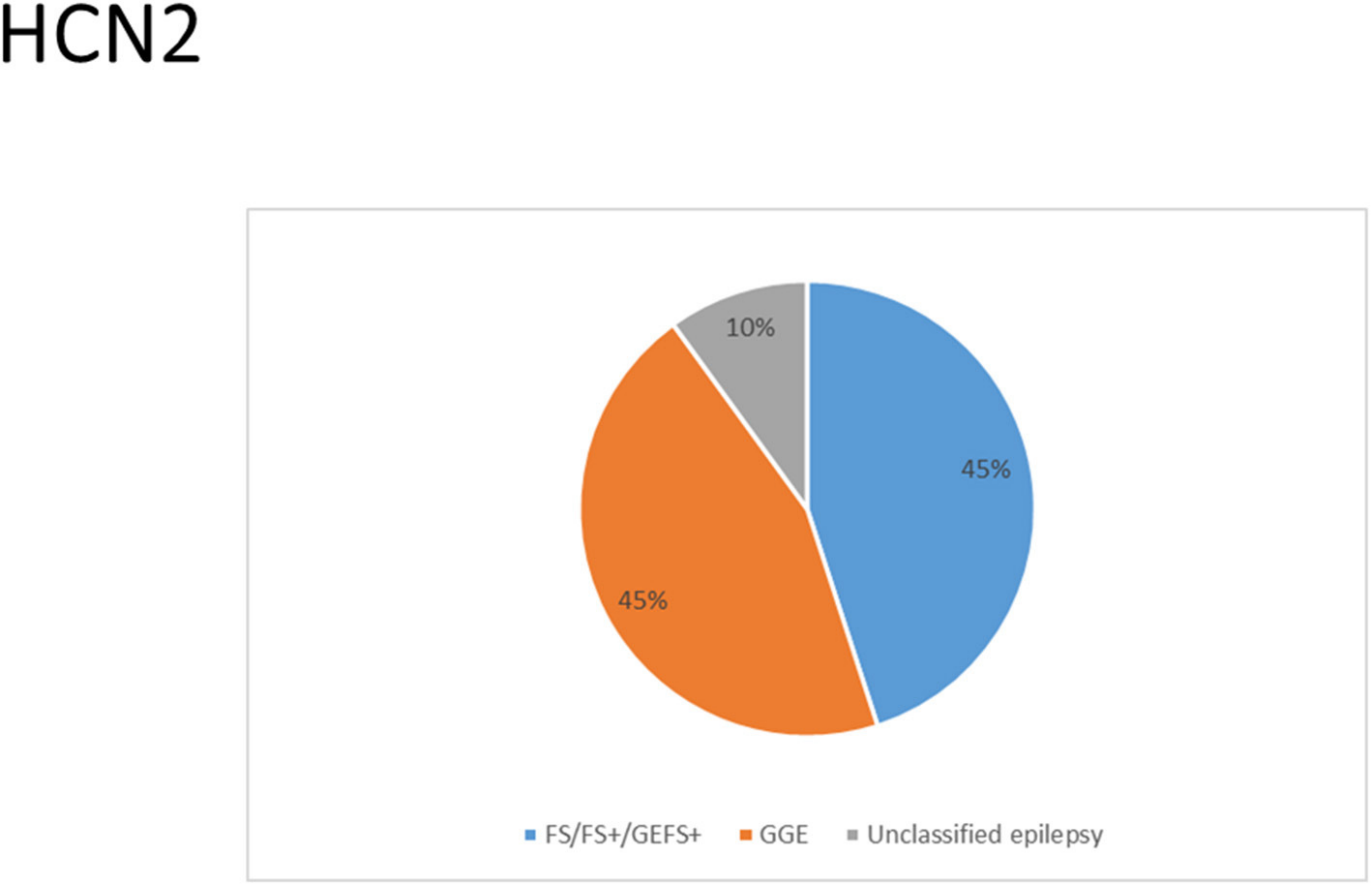
Clinical phenotypes related to *HCN2* variants. Most cases were diagnosed with either febrile seizures (FS), or febrile seizure plus (FS+) or genetic generalized epilepsy with febrile seizure plus (GEFS+) or genetic or idiopathic generalized epilepsy. Cases with unclassed epileptic syndromes include two cases who died due to SUDEP.

**Figure 8:**
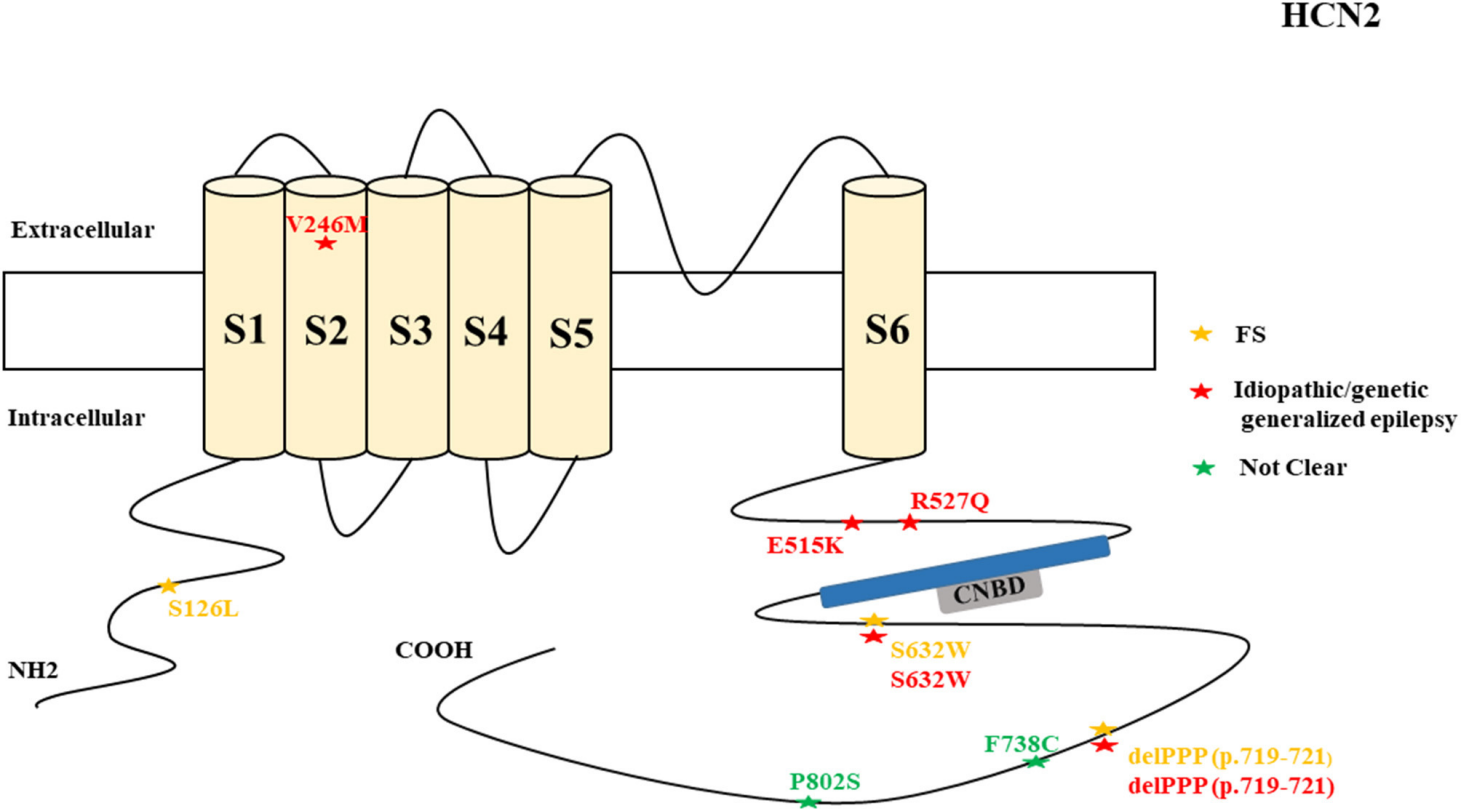
Schematic representation of *HCN2* variants related to different clinical epileptic phenotypes. The p.S632W and delPPP (p. 719–721) are individually related to both febrile seizures and genetic or idiopathic generalized epilepsy. FS stands for febrile seizures and EIEE for early infantile epileptic encephalopathy.

**Figure 9:**
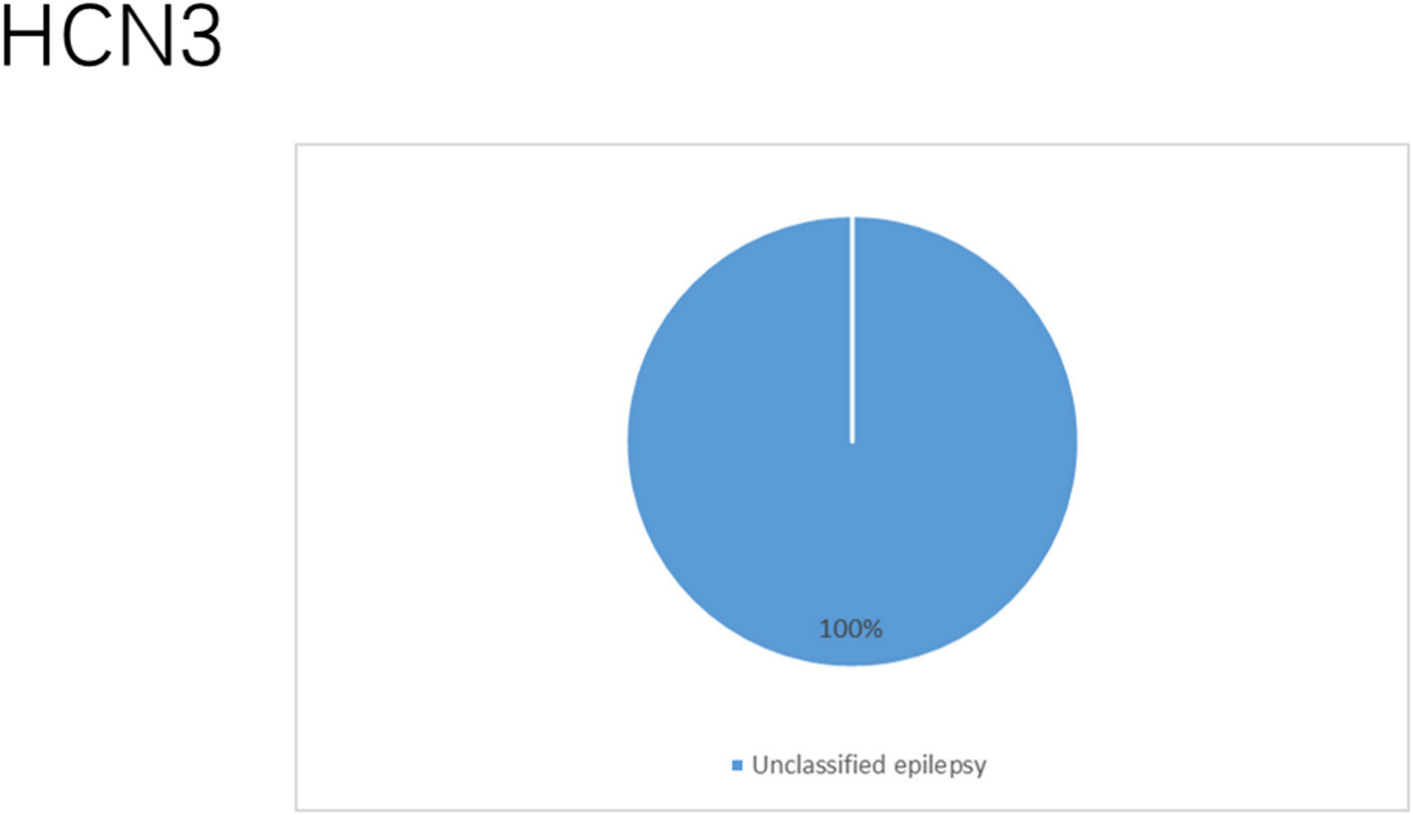
Clinical phenotypes related to *HCN3* variants. All reported cases had unclassified or unknown epileptic syndromes. These are the two cases who died due to SUDEP.

**Figure 10:**
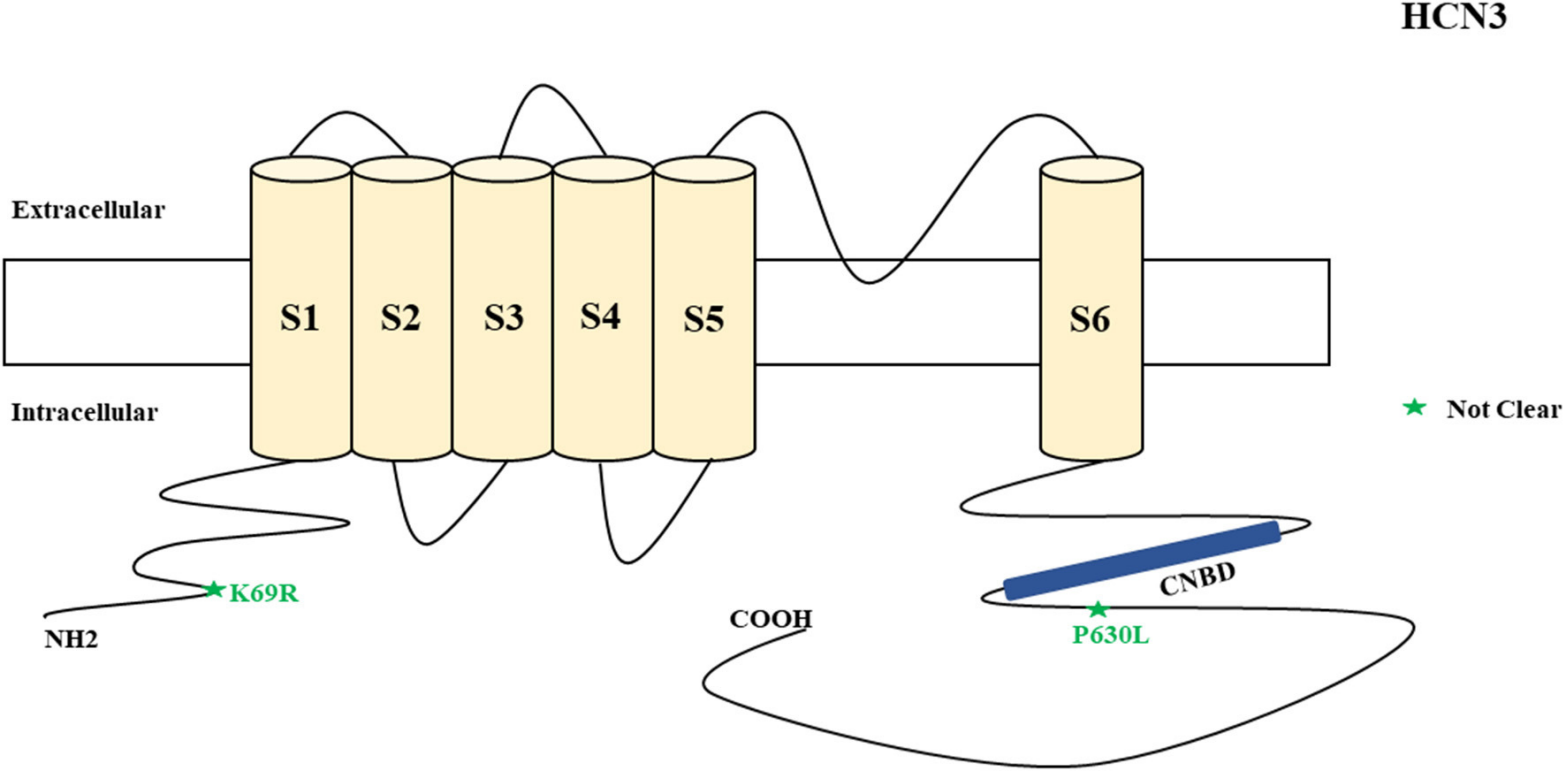
Schematic representation of *HCN3* variants which relate to unclassified or unknown epileptic syndromes.

**Figure 11:**
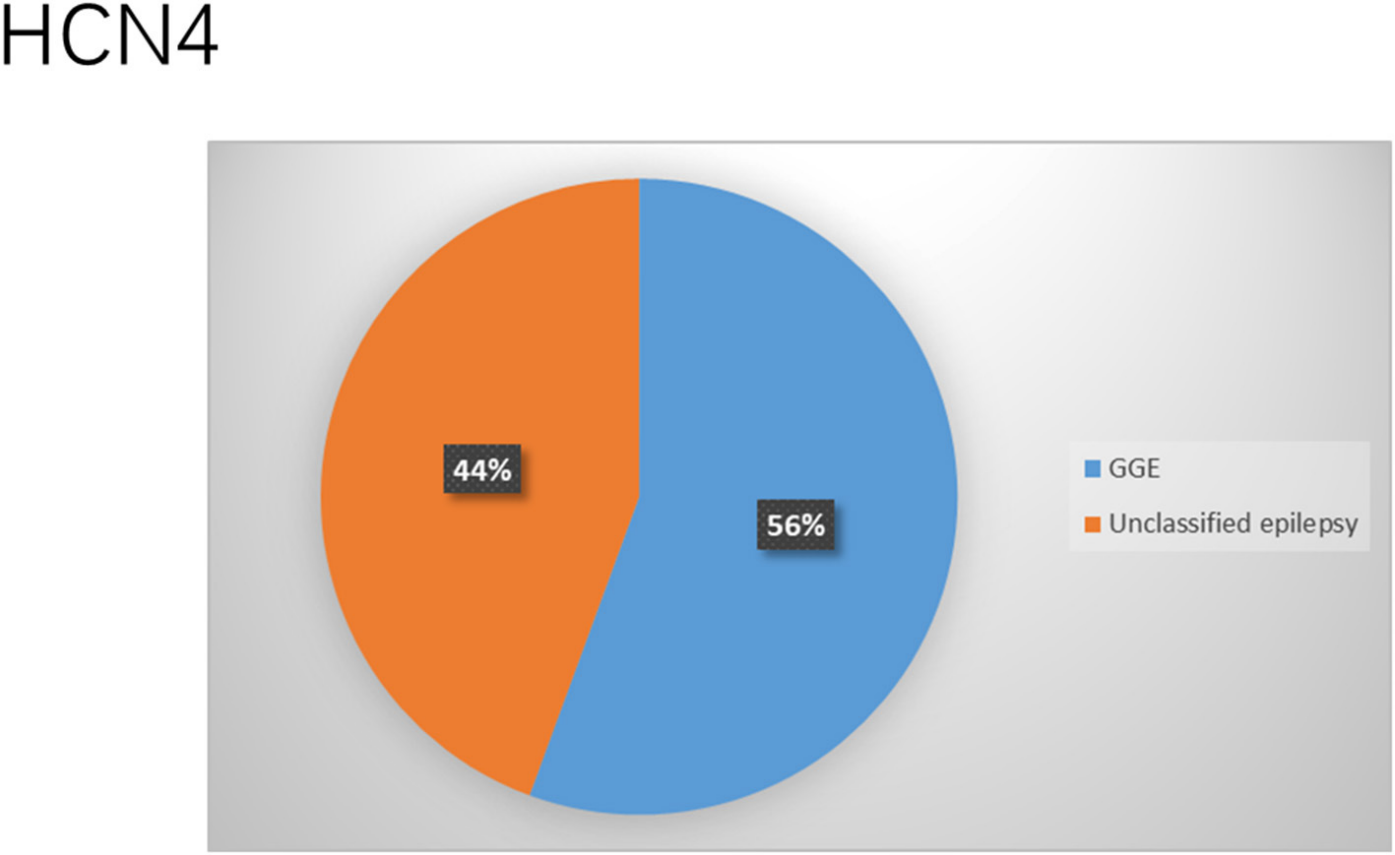
Clinical phenotypes related to *HCN4* variants. Most of the cases were diagnosed with genetic or idiopathic generalized epilepsy (GGE) followed by those with unclassified or unknown epileptic syndrome (majority are those who died due to SUDEP).

**Figure 12:**
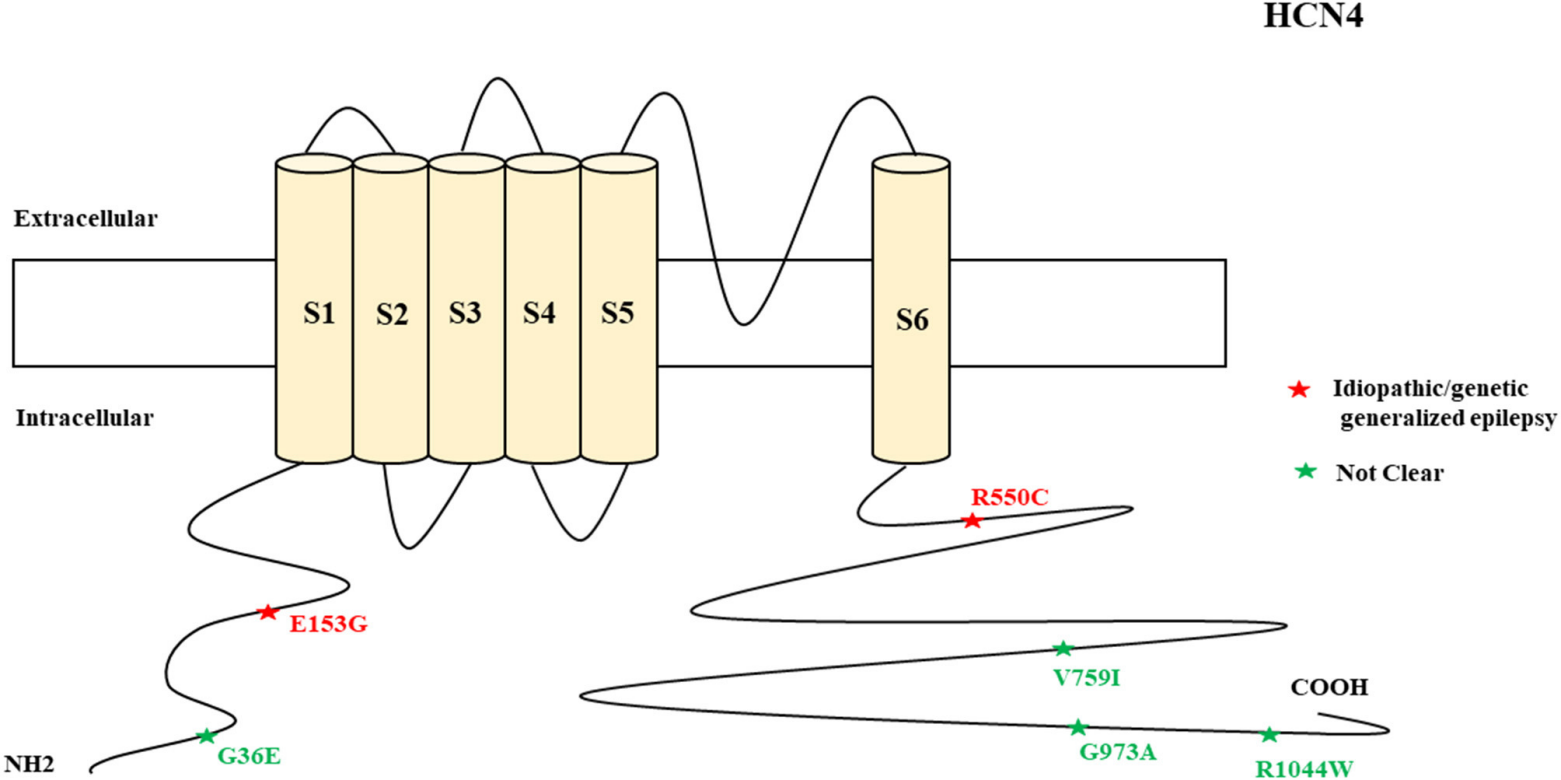
Schematic representation of *HCN4* variants related to different clinical epileptic phenotypes.

There were several genetic mutations in 4 *HCN* genes that were related to epilepsy in 74 cases, comprising *HCN1* (43 cases), *HCN2* (20 cases), *HCN3* (2 cases), and *HCN4* (9 cases) Supplementary Table S1 in Supplementary Material. Both GOF and LOF variants were found in *HCN1* and *HCN2* genes (Figures 1, 2), with unknown functional effects for the *HCN3* gene (Figure 3), and only LOF variants were reported in *HCN4* genes (Figure 4). For the *HCN1* gene, 37.2% (16) of the 43 cases were diagnosed with either febrile seizures, or febrile seizure plus or genetic generalized epilepsy with febrile seizure plus, 23.3% (10) were diagnosed with genetic or idiopathic generalized epilepsy, 16.3% (7) were diagnosed with early infantile epileptic encephalopathy (EIEE), 11.6% (5) were diagnosed with febrile EIEE, and 11.6% (5) presented with unclassified epileptic syndromes (Figure 5). Interestingly, some of the *HCN1* variants are related to different epileptic syndromes: p.M234R is associated with both typical and atypical febrile seizures, p.C329S and p.V414M are related to both febrile seizures and genetic/idiopathic generalized epilepsy, and p.M153I and p.M305L are each related to both EIEE and unclassified epilepsy which occurs in infants (Figure 6). Among 20 cases carrying *HCN2* gene pathogenic variants, 45% (9) were diagnosed with either febrile seizures, or febrile seizure plus or genetic generalized epilepsy with febrile seizure plus, 45% (9) were diagnosed with genetic or idiopathic generalized epilepsy, and 10% (2) presented with unclassed epileptic syndromes (Figure 7). Noteworthy, p.S632W and delPPP (p.719–721) are each related to both febrile seizures and genetic or idiopathic generalized epilepsy (Figure 8). All carriers of the *HCN3* gene variants had unclassified or unknown epileptic syndrome (Figure 9), and the locations of the variants are shown in Figure 10. For the *HCN4* gene, 56% (5) of the 9 cases were diagnosed with genetic or idiopathic generalized epilepsy whereas 44% (4) had unclassified or unknown epileptic syndrome (Figure 11). The locations of the *HCN4* gene variants that are related to unclassified or unknown epileptic syndrome are shown in Figure 12.

Of those 74 cases, 12 (16.2%) died, comprising *HCN1* (*n* = 4), *HCN2* (*n* = 2), *HCN3* (*n* = 2), and *HCN4* (*n* = 4). A number of ten cases (83%) died due to SUDEP and 2 (16.7%) died due to cardiopulmonary failure. A number of one case died at the age of 14 months, one case died at the age of 15 months, one case died at the age of 23 years, and the rest died at the mean age of 40 years. Table 1 provides a summary of the general information of the HCN1–4 channelopathies in relation to epilepsy. Table 2 summarizes the overview of HCN channel subunits, modulators, and pharmacology. Table 3 summarizes the *HCN* genes that are associated with epilepsy and are available in animal models. The detailed results and discussion can be found in the sections below.

**Table 1:** General information of the HCN1-4 channelopathy in relation to epilepsy based on the available information.

**Characteristics**	**Proportion and percentage (when applicable)/further description**			
	***HCN1*** **(43 cases)**	***HCN2*** **(*n* = 20 cases)**	***HCN3*** **(2 cases)**	***HCN4*** **(9 cases)**
**Age of onset**				
Mean (range)	15.4 (0–84) months.	No enough information	No enough information	No enough information/infantile age
**Sex**			No enough information	
Females	25/41 (61%)	3/9 (33.3%)		1/5 (20%)
Males	16/41 (40%)	6/9 (66.7%)		4/5 (80%)
**Pathogenic variants (mode of inheritance)**			No enough information	
*De novo*	26/41 (63.4%)	-		
Inherited	15/41 (36.6%)	12/20 (60%)		
Sporadic	-	2/20 (10%)		
Unknown	2/41 (4.9%)	8/20 (40%)		
**Altered protein function**			No enough information	
GOF	6/26 (23%)	4/8 (50%)		-
LOF	10/26 (38.5%)	1/8 (12.5%)		2/6 (26.7%)
Unknown	10/26 (38.5%)	3/8 (37.5%)		4/6 (66.7%)
**Initial seizure semiology**			No enough information	
Febrile seizures	26/42 (61.9%)	3		-
Tonic seizures	10/42 (23.8%)	-		-
Clonic seizures	5/42 (11.9%)	-		-
Generalized seizures	1/42 (2.4%)	-		3/9 (33.3%)
Absence seizures	2/42 (4.8%)	2		-
**The presence of status epilepticus**			No enough information	No enough information
Yes	5/9 (55.6%)	-		
**Epileptic syndromes**	Childhood focal epilepsy, childhood absence epilepsy, early infantile epileptic encephalopathy (EIEE), febrile seizures, febrile seizure plus, fever-sensitive EIEEs, genetic generalized epilepsy, generalized epilepsy, genetic epilepsy with febrile seizure plus, generalized epilepsy with eyelid myoclonus, neonatal-onset epileptic encephalopathy (MMPSI), and unclassified epilepsy infantile.	Absence seizures, febrile seizures, generalized epilepsy, focal seizures, genetic epilepsy with febrile seizure plus, juvenile myoclonic epilepsy, idiopathic generalized epilepsy, idiopathic photosensitive occipital epilepsy, and photosensitive genetic generalized epilepsy	No enough information	Familial benign myoclonic epilepsy and genetic generalized epilepsy are the major epileptic syndromes
**Additional clinical phenotypes**			No enough information	No enough information
ID/GDD	22/43 (51.1%)	1/20 (5%)		
Others	Behavioral disturbances, autistic features, polyphagia, motor delay, and attention-deficit/hyperactivity disorder, truncal ataxia, language delay, and microcephaly.	Attention-deficit/hyperactivity disorder and abnormal behavior		
**Seizure outcome**				
Seizure free	16/41 (39%)	-	-	2/9 (22.2%)
Controlled seizures	2/41 (4.9%)	-	-	-
Drug-resistant epilepsy	5/41 (12.2%)	1/20 (5%)	-	-
Daily seizures	6/41 (14.6%)	-	-	-
Weekly seizures	1/41 (2.4%)	-	-	-
Rare seizures	3/41 (7.3%)	-	-	-
Monthly seizures,	2/41 (4.9%)	-	-	-
Yearly seizures	2/41 (4.9%)	-	-	-
Died	4/41 (9.8%)	2/20 (10%)	2/2 (100%)	4/9 (44.4%)
Unknown	2/41 (4.9%)	17/20 (85%)	-	3/9 (33.3%)

**Table 2:** An overview of HCN channel subunits, modulators, and pharmacology.

**Gene**	**OMIM number**	**Name**	**Modulators**	**Pharmacology**
*HCN1*	602780	Hyperpolarization-activated cyclic nucleotide-gated potassium channel 1	cAMP (Kanyshkova et al., 2009), casein kinase 2 (Schulze et al., 2020), glycosylation (Zha et al., 2008), protein kinase C (Williams et al., 2015), and phosphorylation (Concepcion et al., 2021)	Blockers include Ivabradine (Bucchi et al., 2006) and MEL55A (Dini et al., 2018), MEL57A (Resta et al., 2018), lidocaine (Putrenko et al., 2017), capsazepine (Gill et al., 2004), ketamine (Zhou et al., 2013), carvedilol (Cao et al., 2018), loperamide, CP-339,818, DK-AH269, and ZD7288 (Lee et al., 2008), and dexmedetomidine (Yang et al., 2014). Nitric oxide suppresses fast Ih current (Kopp-Scheinpflug et al., 2015). CRISPRi or RNA interference (RNAi) (Deutsch et al., 2021) and L-stepholidine reduces HCN1 expression (Zhou et al., 2019a). Cyclophosphamide increased its expression (Liu et al., 2017).
*HCN2*	602781	Hyperpolarization-activated cyclic nucleotide-gated potassium and sodium channel 2	Intracellular chloride ions, pH, cAMP (Kanyshkova et al., 2009), Shox2 (Yu et al., 2021), glycosylation (Zha et al., 2008), phosphorylation (Concepcion et al., 2021), and SUMOylation (Parker et al., 2016)	Blockers include MEL55A (Dini et al., 2018), carvedilol (Cao et al., 2018), dexmedetomidine (Yang et al., 2014). CRISPRi or RNA interference (RNAi) (Deutsch et al., 2021) reduced HCN2 expression.
*HCN3*	609973	Hyperpolarization-activated cyclic nucleotide-gated potassium channel 3	Casein kinase 2 (Schulze et al., 2020)	Blockers include Cs (1+), ZD7288, and Ivabradine (Mistrík et al., 2005)
*HCN4*	605206	Hyperpolarization-activated cyclic nucleotide-gated potassium channel 4	Shox2 (Yu et al., 2021)	The current can be blocked by Ivabradine (Bucchi et al., 2006), EC18 (Kharouf et al., 2020b), carvedilol (Cao et al., 2018), gabapentin (Tae et al., 2017), DK-AH269 (Lee et al., 2008). CRISPRi or RNA interference (RNAi) (Deutsch et al., 2021) reduced its expression.

**Table 3:** HCN subtypes directly and indirectly related to epilepsy in animal models.

**Gene**	**Animal model for epilepsy**	**Phenotype**	**Key findings**
*HCN1*	KO (Nishitani et al., 2019)	Absence epilepsy (Nishitani et al., 2019)	Reduction of Ih current in the cortical and hippocampal pyramidal neurons, pronounced hyperpolarizing shift of the resting membrane potential, and increased input resistance. Prone to pentylenetetrazol-induced acute convulsions. Showed spontaneous spike-wave discharges and behavioral arrest (Nishitani et al., 2019).
	KO (Saito et al., 2012)	Epileptic seizures (Saito et al., 2012)	Ablation of HCN1 in mice augmented the production of amyloid-β peptide (Aβ) (Saito et al., 2012).
	Adult *HCN1*-null mice (Huang et al., 2009)	Kainic acid-induced seizures (Huang et al., 2009)	Loss of dendritic HCN1 subunits which resulted in the enhanced cortical excitability and the development of epilepsy (Huang et al., 2009).
	GABAAγ2 (R43Q) mouse (Phillips et al., 2014)	Absence epilepsy (Phillips et al., 2014)	Diminished hippocampal HCN1 expression and function as well as spatial learning deficit (Phillips et al., 2014).
	*HCN1*-deficient rats (Nishitani et al., 2020)	Absence seizures, loose muscle tension, and abnormal gait (Nishitani et al., 2020).	HCN1 is involved in motor coordination and muscle strength (Boychuk and Teskey, 2017; Boychuk et al., 2017; Nishitani et al., 2020).
	*HCN1* M294L heterozygous knock-in (HCN1M294L) mouse (Bleakley et al., 2021)	Severe developmental impairment and drug-resistant epilepsy (Bleakley et al., 2021)	The mechanism of epilepsy is continuous cation leak that resulted in hyperexcitability of the layer V somatosensory cortical pyramidal neurons (Bleakley et al., 2021).
*HCN2*	*HCN2*-null mice (Ludwig et al., 2003)	Absence seizures (105)	HCN2-deficient mice demonstrated spontaneous absence seizures. The thalamocortical relay had complete loss of the HCN current thus increased hyperexcitabilty. This was accompanied with dysrhythmia (Ludwig et al., 2003).
	*HCN2* knock-in mouse model (HCN2EA) (Hammelmann et al., 2019).	Absence seizures and learning disability (Hammelmann et al., 2019).	cAMP regulates HCN2 channel (Hammelmann et al., 2019).
*HCN4*	Conditional *HCN4* –KO model (Kharouf et al., 2020a).	Seizures (Kharouf et al., 2020a)	EC18 and HCN4-KO reduced seizure susceptibility (Kharouf et al., 2020a).
	*GSK3β [S9A]* mice (Urbanska et al., 2019)	Kainic acid-induced seizures (Urbanska et al., 2019)	GSK3β regulates HCN4 level and the expression of synaptic AMPA receptors (Urbanska et al., 2019).
*TRIP8b*	*TRIP8b* KO (Heuermann et al., 2016)	Absence seizures (Heuermann et al., 2016)	Decreased HCN channel expression and function in thalamic-projecting cortical layer 5b neurons and thalamic relay neurons. Preserved HCN function in inhibitory neurons of the reticular thalamic nucleus (Heuermann et al., 2016).
	*TRIP8b*-null mice (Huang et al., 2012).	Kainic acid-induced seizures (Huang et al., 2012)	Presynaptic adult cortical HCN channel expression continually diminished following induction of seizures and not dendritic HCN channels. Modulation of the adult presynaptic cortical HCN expression is independent of TRIP8b (Huang et al., 2012).
Others	Genetic Absence Epilepsy Rats from Strasbourg (GAERS) model (Cain et al., 2015)	Absence seizures (Cain et al., 2015)	Increased HCN-1 and HCN-3 expression in ventrobasal thalamic neurons and the blockage of Ih current suppressed burst-firing (usually accompany spike-and-wave discharges) (Cain et al., 2015).
	Genetic Absence Epilepsy Rats from Strasbourg (GAERS) model (Kuisle et al., 2006)	Absence seizures (Kuisle et al., 2006)	The binding of cAMP to HCN channels was weakened in acute phase thus promoted epilepsy and the compensatory mechanisms to stabilize Ih current activity led to the cessation of spike-and-wave discharges in chronic epilepsy. Calcium ions trigger the synthesis of cAMP (Kuisle et al., 2006).
	Genetic Absence Epilepsy Rats from Strasbourg (GAERS) and acquired temporal lobe epilepsy model (Smith and Delisle, 2015)	Absence seizure and status epilepticus (Smith and Delisle, 2015)	Diminished cardiac expression of HCN2 in both models. Chronic epilepsy can induce cardiac channelopathies thus SUDEP (Smith and Delisle, 2015)
	Genetic Absence Epilepsy Rats from Strasbourg (GAERS) and acquired temporal lobe epilepsy (Powell et al., 2014)	Post–status epilepticus (Powell et al., 2014)	Secondary ion channelopathies and cardiac dysfunction can result from the chronic epilepsy (Powell et al., 2014)
	Genetic Absence Epilepsy Rats from Strasbourg (GAERS), male Wistar rats, male Stargazer mice (David et al., 2018)	Absence seizures (David et al., 2018)	Blockage of HCN channels *via* ZD7288 antagonist in ventrobasal thalamus decreases thalamocortical neuron firing and eliminates spontaneous absence seizures in GAERS, Wistar rats and male Stargazer mice (David et al., 2018).
	Wistar Albino Glaxo rats, bred in Rijswijk (Budde et al., 2005)	Absence epilepsy (Budde et al., 2005)	There is a need of the balance of HCN1 and HCN2 gene expression in thalamocortical for the modulation of burst firing in thalamic networks (spindle-like or spike-wave-like patterns). Increased expression of HCN1 and no changes for the rest of HCN channels (Budde et al., 2005).
	Rat Pilocarpine Model of Epilepsy (Jung et al., 2007)	Spontaneous induced recurrent seizures (Jung et al., 2007)	The diminished expression of the dendritic HCN channels during the acute phase of the epilepsy is accompanied by the loss of hyperpolarization of voltage-dependent activation. These phenomena progressed to the chronic phase which increases neuronal excitability and thus epileptogenesis. Phenobarbital could suppress seizures and reversed the current changes but not the expression (Jung et al., 2007).
	Rat Pilocarpine Model of Epilepsy (Jung et al., 2011)	Spontaneous induced recurrent seizures (Jung et al., 2011)	Loss Ih current and HCN1channel expression start 1 h after status epilepticus and involves several steps including dendritic HCN1 channel internalization, deferred loss of protein expression, and finally the downregulation of mRNA expression (Jung et al., 2011).
	Wistar Albino Glaxo/Rij strain (Wemhöner et al., 2015)	Absence epilepsy (Wemhöner et al., 2015)	Gain-of-function of WAG-HCN1is caused by N-terminal deletion, increase of the HCN1expression and current, suppression of HCN2 and HCN4 currents as well as reduction of cAMP sensitivity (Wemhöner et al., 2015).
	Tottering mice (Kase et al., 2012)	Absence seizures (Kase et al., 2012)	Reduction of HCN function which led to enhancement of membrane excitability in subthalamic nucleus neurons. The activation of HCN channel activity *in vitro* could rescue the situation (Kase et al., 2012).

*KO, knockout*.

#### HCN1

*HCN1* encodes for the hyperpolarization-activated cyclic nucleotide-gated potassium channel 1 (HCN1). HCN1 protein is expressed in the hippocampus (Lee et al., 2019), cerebral cortex (Shah, 2014; Zhou et al., 2019b), cerebellum (Luján et al., 2005), thalamus (Kanyshkova et al., 2012), amygdala (Knoll et al., 2016), and entorhinal cortex (Nolan et al., 2007). In the brain, they are further spread in layer 5 of neocortical neurons, CA1 and CA3 hippocampal regions, molecular cell layer of the cerebellum (Gravante et al., 2004), superficial layers (II/III) of the pyramidal neurons (Santoro and Shah, 2020), and parvalbumin-positive interneurons (Notomi and Shigemoto, 2004). In the cerebral cortex, HCN1 channels are primarily located on pyramidal cell dendrites and at lower concentrations in the soma of pyramidal neurons where they regulate spike firing and synaptic potential integration by influencing the membrane resistance and resting membrane potential (Shah, 2014). HCN1 current does modulate excitatory and inhibitory postsynaptic potentials in the cerebral cortex and hippocampal neurons and regulates the tonic firing of cerebellar neurons (Rivolta et al., 2020). Besides, HCN1 channels are expressed in astrocytes (Honsa et al., 2014) and microglia (Vay et al., 2020). *HCN1* pathogenic variants are related to several types of epileptic syndromes (Figure 5). More than half of the cases do present with intellectual disability (ID) or global developmental delay (GDD). Some cases may manifest additional clinical features, such as behavioral disturbances, autistic features, polyphagia, motor delay, attention-deficit hyperactivity disorder (ADHD), truncal ataxia, language delay, and microcephaly. Both GOF and LOF variants are implicated in *HCN1* (Figure 1). It is a major concern that 4 *HCN1* cases died (Table 1). Most of the mutations are located in S6, N-terminal, and the intracellular linker between S6 and CNBD. Notably, variants that are related to SUDEP are located both in the N- and C-terminals, and there are hotspots in residue G391 (Figure 1).

Epilepsies are more likely to result from a loss of the inhibitory component than from a gain of the excitatory component. Therefore, it is expected that only LOF mutations (loss of the inhibitory component in interneurons) can result in epilepsy. Interestingly, GOF variants can also produce epilepsy, and this suggests the existence of other unknown mechanisms. We hypothesize that the neuroanatomical localization of the HCN pathogenic variants either on principal or pyramidal neurons or on inhibitory interneurons along with their distribution patterns either in the dendrites, axons, or soma can possibly explain the underlying mechanisms for epilepsy for both GOF and LOF variants. Unfortunately, most of the functional studies performed for the reported variants were limited to the electrophysiological studies, and only a few were done to explore the neuroanatomical localization in different neuronal subtypes not to mention the distribution patterns.

Fever-sensitive EIEE can be caused by both GOF (p.S100F, p.D401H, and p.H279Y) and LOF (p.S272P, and p.R297T) HCN1 pathogenic variants, and the outcome is poor (Nava et al., 2014). A number of two cases from different studies with the same pathogenic variant (p.L157V) with LOF (dominant-negative effect) effect on the electrophysiological studies carried out on Chinese hamster ovary (CHO) cells and neonatal rat cortical neurons, presented with the same clinical phenotype of genetic generalized epilepsy, although the outcome was unclear for one case (Bonzanni et al., 2018; DiFrancesco et al., 2019). The LOF effect of p.L157V on neonatal rat cortical neurons increased neuronal excitability and discharge activity, hypothetically related to epileptogenesis. The effect of this mutation on cortical neurons seems to be similar to pyramidal neurons; however, further experiments are needed to know whether the loss of Ih current for this particular variant can also eliminate dendritic attenuation as in pyramidal neurons (Noam et al., 2011). A total of two cases carrying the GOF variant (p.M153I) according to the electrophysiological study on CHO-K1 cells presented with two different epileptic syndromes, namely, EIEE and unclassified epilepsy of infantile-onset. Then, one of them had daily seizures and the other one had weekly seizures, which suggests that one variant can present with different phenotypes (Marini et al., 2018). A total of two cases carrying the LOF variant (p.M243R) according to the electrophysiological study on CHO-K1 cells presented with febrile seizures and febrile seizure plus, and they also manifested as rare seizures (Marini et al., 2018).

A total of two cases carrying the LOF variant (p.M305L) based on the electrophysiological studies performed on both CHO-K1 and HEK293T cells presented with two different phenotypes with different severities, which suggests that the same mutation can produce different phenotypes. A number of one case presented with unclassified infantile epilepsy and mild GDD, and the patient achieved seizure freedom. The other case presented with EIEE, severe ID, and microcephaly, and this patient also achieved seizure freedom (Marini et al., 2018). The exploration of the neuroanatomical localization and distribution pattern of this variant is needed. The variant of p.R590Q (LOF according to the electrophysiological study on CHO-K1 cells) was associated with childhood absence seizures in two separate case reports (Marini et al., 2018; DiFrancesco et al., 2019). This variant affects a residue of CNBD which is critical for cAMP binding. However, the two cases that were reported had mild phenotypes.

The residue G391 seems to be a hotspot and is associated with both mild and severe phenotypes. The p.G391S and p.G391C are related to milder phenotypes and are associated with GOF and LOF effects, respectively, when co-transfected with the wild-type according to the electrophysiological studies performed on both CHO-K1 and HEK293T cells. In contrast, p.G391D with LOF effect on the electrophysiological studies performed on both CHO-K1 and HEK293T cells is resulted in the most severe phenotype which was associated with 2 deaths (Marini et al., 2018). This implies that the severity of the phenotype may depend on the type of the variants rather than the location of the variants. Notably, it is difficult to understand the underlying mechanisms of these variants since functional experiments were only limited to the electrophysiological studies. Some of the reported *HCN1* pathogenic variants, such as p.M305L, p.G391D, and p.S399P, demonstrated no current on electrophysiological studies conducted on both CHO-K1 and HEK293T cells, which implies LOF effect (Marini et al., 2018). For p.M305L which is located in the S5 domain, it has been shown recently that there is a loss of voltage dependence in the activation and deactivation according to the electrophysiological study performed on Xenopus oocytes, leading to continuous excitatory cation flow at membrane potentials that would usually close the channel (Hung et al., 2021).

Two more cases died in other studies, and the postmortem results showed that one carried the non-synonymous novel p.G46V (Tu et al., 2011) and the other one carried a variant of unknown significance, p.72_74del (c.187_195delGGCGGTGGC) (Coll et al., 2016). Coll et al. (2016) performed a custom resequencing panel, including 9 genes known to be involved in SUDEP and 88 candidate genes among 14 SUDEP cases from both postmortem (2 Cases) and from alive patients (12 Cases), in which they found this variant [p.72_74del (c.187_195delGGCGGTGGC)]. Likewise, Tu et al. (2011) performed a genome-wide association study to investigate the role of pathogenic DNA variants in the *HCN1–4* genes in a large SUDEP cohort involving 48 SUDEP cases in which they identified six novels and three previously reported non-synonymous variants, including p.G46V. The former 2 cases (carrying p.G391D variant) died due to cardiopulmonary failure and the latter (carrying p.G46V and p.72_74del (c.187_195delGGCGGTGGC variants) died due to SUDEP. Two of the variants that were associated with deaths are clustered in the N-terminal part of the channel, and one variant is in the C-terminal. HCN1 channels play a role in the enhancement of long-term potentiation, synaptic plasticity, and cognitive process (Beaumont et al., 2002; Zhong and Zucker, 2004). Therefore, their dysfunction can result in neurodevelopmental disorders, such as ID, GDD, and ADHD. Noteworthy, some of the same *HCN1* variants are related to different epileptic syndromes: p.M234R is associated with both typical and atypical febrile seizures, p.C329S and p.V414M are each related to both febrile seizures and genetic or idiopathic generalized epilepsy, and p.M153I and p.M305L are each related to both EIEE and unclassified epilepsy which occurs in infants (Figure 6). This suggests heterogeneity of the *HCN1* phenotypes. In summary, based on the electrophysiological studies on cell models, both GOF and LOF are associated with epilepsy; but LOF is associated with more severe phenotypes, including 3 deaths due to the deletion and strong reduction in HCN1 current density. The underlying mechanisms for both GOF and LOF variants remain unclear since most of the functional studies were limited to the electrophysiological studies, and only a few were done to explore the neuroanatomical localization in different neuronal subtypes not to mention the distribution patterns.

Most rodent models of epilepsy support the fact that the loss of the HCN1 current in pyramidal, cortical, and thalamic neurons is associated with the occurrence of epilepsy, but there is limited evidence showing that the upregulation of this current can produce epilepsy. The HCN1-knockout rat model of the absence seizures reveals a reduction of Ih current in the cortical and hippocampal pyramidal neurons, pronounced hyperpolarizing shift of the resting membrane potential, and increased input resistance. Besides, this model is prone to pentylenetetrazol-induced acute convulsions and shows spontaneous spike-wave discharges and behavioral arrest (Nishitani et al., 2019). The loss of dendritic HCN1 subunits in entorhinal cortical and hippocampal pyramidal cell dendrites leads to the enhancement of cortical excitability and greater seizure susceptibility in adult HCN1-null mice (Huang et al., 2009). *In vitro* electrophysiological studies disclosed that this greater seizure susceptibility of adult HCN1-null mice occurs due to enhanced excitability of entorhinal cortical layer III neurons as a result of lack of dendritic Ih current. Therefore, the lack of dendritic Ih current in pyramidal cell results in an imbalance in excitatory and inhibitory synaptic activity which influence cortical neural network activity (Huang et al., 2009). Altogether, these studies suggest that the reduction of HCN1current in the neocortex and hippocampus can lead to the absence epilepsy. The HCN1 p.M294L heterozygous knock-in (HCN1M294L) mouse demonstrates the clinical manifestations of patients with the *HCN1* p.M305L variant, including spontaneous seizures, learning deficit, seizure exaggeration by lamotrigine, and the seizure reduction by sodium valproate (Bleakley et al., 2021). The functional analysis of HCN1M294L on Xenopus laevis oocytes and layer V somatosensory cortical pyramidal neurons in *ex vivo* tissue revealed a loss of voltage dependence that was accompanied by open channel that allowed for cation “leak” resulting in layer V somatosensory cortical pyramidal neurons and CA1 hippocampal pyramidal neuron depolarization at rest (Bleakley et al., 2021). Therefore, impaired voltage-dependent gating properties of HCN1 channels due to certain variants can allow continuous excitatory cation flow that produces epilepsy (Bleakley et al., 2021; Hung et al., 2021).

The GOF property of WAG-HCN1 (animal model of absence seizures) is caused by N-terminal deletion, change in N-terminal wild-type sequence (GNSVCF) motif, increased current, enhanced HCN1 expression, reduced cAMP sensitivity, and suppressed HCN2 and HCN4 currents according to the functional analysis performed on Xenopus oocytes and thalamus of WAG/Rij rat strain (Wemhöner et al., 2015). The Genetic Absence Epilepsy Rats from Strasbourg (GAERS) is a model of absence seizures. It reveals an increase of HCN-1 and HCN-3 expression in the ventrobasal thalamic neurons (subsets of thalamic relay neurons) and enhanced Ih current which suppresses neuronal burst firing (Cain et al., 2015). This model also shows a reduction of T-type calcium channel whole-cell currents in ventrobasal thalamic, CaV3.1 mRNA, and protein levels (Cain et al., 2015). The Wistar Albino Glaxo rat that is bred in Rijswijk (WAG/Rij) is an animal model of absence seizures. It shows the need for the balance of *HCN1* and *HCN2* gene expression in the thalamocortical area, to enable the modulation of burst firing in thalamic networks (Budde et al., 2005). Notably, there is an increased expression of HCN1 on mRNA and protein levels and no further changes to the other HCN channels' expression (Budde et al., 2005). The tottering mice of absence seizures is a model that demonstrates the reduction of HCN function and the resultant enhancement of membrane excitability in subthalamic nucleus neurons, although the activation of HCN channel activity *in vitro* can reverse the situation (Kase et al., 2012). Thus, based on these studies, it seems that enhanced Ih current in the thalamus can suppress neuronal burst firing in the absence epilepsy. In addition, the reduction of the T-type calcium channel currents in the ventrobasal thalamus can play a role in suppressing neuronal burst firing in the absence epilepsy.

The rat pilocarpine model of epilepsy is an animal model of spontaneous, induced, and recurrent seizures. It revealed that there is a diminished expression of the dendritic HCN channels during the acute phase of the epilepsy, and this is accompanied by the loss of channel expression and hyperpolarization of voltage-dependent activation (Jung et al., 2007). This change may progress to the chronic phase which increases neuronal excitability and thus epileptogenesis (Jung et al., 2007). Phenobarbital can suppress seizures and reverse the neuronal current changes, but not the expression of HCN channel (Jung et al., 2007). The loss of Ih current and HCN1 channel expression starts 1 h after status epilepticus: it involves several steps, including dendritic HCN1 channel internalization, deferred loss of protein expression, and the final downregulation of mRNA expression (Jung et al., 2011).

There are currently some HCN1 channel blocker drugs, but they have low efficacy. MEL55A is a potential HCN1/2 blocker, but it may also increase seizure susceptibility (Kharouf et al., 2020b). However, another study showed that MEL55A does not affect seizure susceptibility (Kharouf et al., 2020b). This ambiguity could be due to the low binding affinity of MEL55A. A recent study of HCN1 channels unveiled the hidden hydrophobic groove in the pore that may be responsible for the low binding affinity (Tanguay et al., 2019). Table 3 summarizes this information. New HCN1-blocking drugs with high affinity may be developed in the future. Since HCN1 current is essential for the prevention of epilepsy, there is also the need to develop HCN1 channel openers.

#### HCN2

*HCN2* encodes for the hyperpolarization-activated cyclic nucleotide-gated potassium channel 2 (HCN2). HCN2 channels are expressed in the hippocampus (Lee et al., 2019), cerebral cortex (Shah, 2014), cerebellar Purkinje cells (Han et al., 2002), thalamus (Kanyshkova et al., 2012), cholinergic interneurons of nucleus accumbens (Cheng et al., 2019), and hippocampal inhibitory interneurons (Matt et al., 2011). They are also expressed in astrocytes (Honsa et al., 2014), microglia (Vay et al., 2020), and oligodendrocytes (Notomi and Shigemoto, 2004; Swire et al., 2021). In the cerebral cortex, HCN2 channels are primarily located on pyramidal cell dendrites and at lower concentrations in the soma of pyramidal neurons where they regulate spike firing and synaptic potential integration by influencing the membrane resistance and resting membrane potential (Shah, 2014). HCN2 channels in hippocampal inhibitory interneurons modulate synaptic plasticity by enabling the GABAergic output onto pyramidal neurons (Matt et al., 2011).

*HCN2* pathogenic variants are associated with absence seizures, febrile seizures, generalized epilepsy, focal seizures, genetic epilepsy with febrile seizure plus, juvenile myoclonic epilepsy, idiopathic generalized epilepsy, idiopathic photosensitive occipital epilepsy, and photosensitive genetic generalized epilepsy (Figure 7). Of the 8 reported *HCN2* variant cases, 4 were GOF and 1 was LOF according to the electrophysiological studies (Figure 2). Then, two cases died. It is surprising that most of the variants had a GOF effect rather than LOF, which is in contrast to HCN1 variants. Similar to HCN1, some of variants are related to different epileptic syndromes; p.S632W and delPPP (p.719–721) are each related to both febrile seizures and genetic or idiopathic generalized epilepsy (Figure 8).

The *HCN2* cases with GOF variants according to the electrophysiological studies performed on oocytes from Xenopus laevis include the inherited p.S632W variant as observed in 2 cases with idiopathic photosensitive occipital epilepsy, 1 case with febrile seizures, and 1 case with absence seizures. It included the inherited p.V246M variant as identified in 1 case with photosensitive generalized genetic epilepsy, 1 case with juvenile myoclonic epilepsy, and 1 case with generalized or focal seizures (Li et al., 2018). The GOF effects of these variants resulted in a depolarized resting membrane potential that took neurons closer to a threshold for action potential firing (Li et al., 2018). The inherited p.S126L variant with the GOF property according to the electrophysiological study on HEK293 cells was detected in 2 cases with febrile seizures (Nakamura et al., 2013). Electrophysiological study of the *HCN2* variant p.S126L (with GOF effect) revealed substantial cAMP-independent enhanced availability of Ih currents during high temperatures, which can explain hyperthermia-induced neuronal hyperexcitability in some cases with febrile seizures (Nakamura et al., 2013). It has been shown that hyperthermia may reduce GABAA receptor-mediated synaptic inhibition in hippocampal CA1 neurons of immature rats (Qu et al., 2007). Therefore, the decrease of the GABAA receptor-mediated synaptic inhibition which is coupled with the shift in the activation kinetics of *HCN2* p.S126L during hyperthermia can accelerate the development of the febrile seizures. Thomas et al. (2019) performed a computational or simulation study on hippocampal CA1 pyramidal neuron synapse model to explore the effect of three *HCN2* variants with GOF property reported before (p.S126L, p.S632W, and p.V246M). Their study unveiled that for the GOF variants to cause neuronal hyperexcitability, the depolarizing effect of HCN2 currents must be greater than the effects of decreased input resistance (Thomas et al., 2019). The variant delPPP (p.719–721) with the GOF property according to the electrophysiological study on oocytes from Xenopus laevis was detected in the 6 cases that presented with febrile seizures and genetic epilepsy with febrile seizure plus (Dibbens et al., 2010). Similarly, the increase of the depolarizing membrane potential that takes neurons closer to the firing potential has been proposed as a possible mechanism of epilepsy for delPPP (p.719–721) (Dibbens et al., 2010). The *HCN2* knock-in mouse model (HCN2EA) in which the tie of cAMP to HCN2 was abolished by two variants (p.R591E and p. T592A) showed that cAMP gating is vital for the regulation of the transition between the burst and tonic firing in thalamic dorsal lateral geniculate and ventrobasal nuclei (Hammelmann et al., 2019). *HCN2EA* mice exhibited generalized seizures of thalamic origin (Hammelmann et al., 2019). This suggests that cAMP regulates the HCN2 channels and the abolishment of cAMP sensitivity in HCN2 channels produces generalized seizures (Hammelmann et al., 2019).

The LOF variants based on the electrophysiological studies on CHO cells and neonatal rat cortical neurons include the sporadic heterozygous p.E515K as identified in 2 cases who presented with generalized epilepsy (DiFrancesco et al., 2011, 2019). The p.E515K variant causes increased neuronal excitability in newborn rat cortical neurons. Thomas et al. (2019) performed a computational or simulation study on a hippocampal CA1 pyramidal neuron synapse model to explore the effect of *HCN2* p. E515K variant. Their study unveiled that for the LOF variants to cause neuronal hyperexcitability, the increased input resistance must be greater than the hyperpolarization in resting membrane potential due to the low levels of Ih (Thomas et al., 2019). It has been shown that HCN2 channels drive inhibitory signal from local interneurons onto distal dendrites of CA1 pyramidal neurons, and a loss of these channels on interneurons impairs inhibition of CA1 pyramidal cells in mice carrying a global deletion of the channel (HCN2–/–) (Matt et al., 2011). Two non-synonymous novel variants, F738C and P802S, were identified in two cases in postmortem (Tu et al., 2011). Notably, these variants are located in the C-terminal of the channel. The HCN2 p.R527Q variant of unknown significance has been reported in one case that presented with idiopathic generalized epilepsy (Tang et al., 2008).

The *HCN2*-null mouse demonstrates a complete loss of the Ih current in thalamocortical relay neurons, leading to increased neuronal hyperexcitability (spontaneous absence seizures) and dysrhythmia (Ludwig et al., 2003). The suggested mechanism for the occurrence of the absence seizures includes the hyperpolarizing shift in the resting potential of HCN2-deficient thalamocortical relay neurons, which eradicates inactivation from T-type Ca2+ channels and thus stimulates low-threshold burst firing in response to depolarizing inputs (Ludwig et al., 2003). This implies that the loss of inhibitory components in thalamocortical relay neurons can explain the occurrence of epilepsy. Dysrhythmia and cardiac dysfunction due to autonomic disturbances may be the possible cause of SUDEP. Compound 4e can inhibit the HCN2 channel (Chen et al., 2019). MEL55A is a potential HCN1/2 blocker but it increases seizure susceptibility (Kharouf et al., 2020b). Propofol can block HCN2 current, leading to the reduction of neuronal excitability and burst firing in thalamocortical neurons, *in vivo* and *in vitro* (Ying et al., 2006). Currently, there is no pharmacological agent that opens the HCN2 channel.

#### HCN3

*HCN3* encodes for the hyperpolarization-activated cyclic nucleotide-gated potassium channel 3 (HCN3). HCN3 channels are found in the cerebellum (Zúñiga et al., 2016), supraoptic nucleus of hypothalamus (Monteggia et al., 2000), thalamus (Kanyshkova et al., 2012), and Martinotti cells (somatostatin-expressing interneurons) (Wang et al., 2004). Martinotti cells are located in different neocortical layers where they provide feedback inhibition in and between neocortical layers and columns (Wang et al., 2004). HCN3 channels are also expressed in astrocytes (Honsa et al., 2014) and microglia (Vay et al., 2020). Non-synonymous variants of *HCN3* have been described in humans and are associated with epilepsy and SUDEP (Figure 3). Such variants (p.K69R and p.P630L) were identified in two cases in postmortem (Tu et al., 2011). However, there was inadequate information regarding their functional properties (Figure 3) and clinical phenotypes. Notably, p.K69R is located in the N-terminal whereas p.P630L is in the C-terminal. There is no HCN3 animal model for epilepsy. A study showed that *HCN3*-deficient mice exhibit compromised processing of contextual information, but this study only explored the role of HCN3 channel in the regulation of circadian rhythm and behavior, rather than epilepsy (Stieglitz et al., 2018). Due to the fact that HCN3 channelopathy is related to epilepsy and SUDEP, future studies can explore its association with the occurrence of epilepsy.

Ivabradine can block HCN3 current in HEK293T cells (Mistrík et al., 2005). It is an approved drug for the treatment of systolic heart failure and chronic stable angina (Koruth et al., 2017), and it has shown anticonvulsant effects in epileptic animal models. It has anticonvulsant, neuroprotective, and antioxidant effects, especially against pentylenetetrazole-induced and picrotoxin-induced seizures in mice (Cavalcante et al., 2019). It acts through GABA-A receptors (Cavalcante et al., 2019). It is more effective in models of febrile or thermogenic seizures (Kharouf et al., 2020b), electroshock-induced tonic seizures in mice (Luszczki et al., 2013), and absence seizures when administered orally (Iacone et al., 2021). Ivabradine can reduce the potency of lamotrigine in epilepsy patients, as shown in mice. However, it can be co-administered with lacosamide, pregabalin, and topiramate (Sawicka et al., 2017). Sodium valproate, gabapentin, and carbamazepine are effective in blocking epileptic activities in the hippocampus that occur among others due to magnesium or potassium ion dysfunction (Arias and Bowlby, 2005). Table 3 summarizes this information.

#### HCN4

*HCN4* encodes for the hyperpolarization-activated cyclic nucleotide-gated potassium channel 4. HCN4 channels are distributed in the neocortex (Battefeld et al., 2012), cerebellum (Zúñiga et al., 2016), hippocampus and spinal cord (Hughes et al., 2013; Nakagawa et al., 2020), cerebellar Purkinje fibers (Han et al., 2002), thalamus (Kanyshkova et al., 2012; Oyrer et al., 2019), corpus striatum, globus pallidus, and habenula (Oyrer et al., 2019). They can be found in neurons that regulate spontaneous rhythmic activity, such as in the thalamocortical relay, substantia nigra, cholinergic interneurons, and medial habenula (Santoro et al., 2000; Notomi and Shigemoto, 2004). Additionally, they can also be found in fast-spiking interneurons of the rat hippocampus (Hughes et al., 2013) and also in the astrocytes (Honsa et al., 2014) and microglia (Vay et al., 2020). HCN4 channels are activated with hyperpolarizing potentials: therefore, they are involved in the neuronal hyperexcitability that is observed in seizures (Sartiani et al., 2017).

The clinical features of HCN4 channelopathy manifest in infantile age, as shown in two cases (Campostrini et al., 2018). Idiopathic or genetic generalized epilepsy, including familial benign myoclonic epilepsy and genetic generalized epilepsy, are the major epileptic syndromes (Figure 11). Two variants are LOF (Figure 4), whereas 4 variants have not been characterized. The familial heterozygous variant p.R550C (LOF) was observed in 4 cases with familial benign myoclonic epilepsy (Campostrini et al., 2018; DiFrancesco et al., 2019) and inherited variant p.E153G in a case with genetic generalized epilepsy (Becker et al., 2017). The p.R550C variant increased neuronal excitability in CHO cells and pyramidal neurons according to the electrophysiological analysis findings (Campostrini et al., 2018). This implies that the loss of Ih current in pyramidal neurons can produce epilepsy.

Four non-synonymous novel variants, p.G36E, p.V759I, p.G973R, and p.R1044W, were identified in two *HCN4* cases in postmortem (Tu et al., 2011). Three of these variants are located in the C-terminal of the channel. Among the four *HCN* genes, *HCN4* has the highest mortality rate and the reason remains unclear. However, future studies can explore the possible reasons. *HCN4*-knockout mice showed that thalamic ventrobasal nuclei-specific deletion of *HCN4* cannot induce generalized seizures of the absence type (Hammelmann et al., 2019). Another study showed that brain-specific *HCN4*-knockout adult mice exhibit reduced seizure susceptibility in cortical neurons; this suggests that HCN4 channel suppression may decrease seizure susceptibility and neuronal excitability (Kharouf et al., 2020a). The study concluded that HCN4 channels are the controllers of brain excitability since their inhibition reduces thalamocortical bursting firing, and that blockers of these channels can be effective antiseizure medications (Kharouf et al., 2020a). This implies that increased HCN4 current can produce seizures. This is also supported by the evidence of the augmented HCN4 mRNA levels in the pilocarpine rodent model of temporal lobe epilepsy that relates to enlarged Ih in dentate granule cells (Surges et al., 2012). Since the reported *HCN4* cases had epilepsy and pathogenic variants with LOF effects, there is a need to investigate how LOF mutations can produce seizures. One of the possible reasons is the loss of Ih current in pyramidal neurons that can produce epilepsy; in contrast, the loss of Ih current in thalamocortical neurons is protective against epilepsy. *HCN4*-knockout mice with dorsal hippocampus dysfunction exhibit anxiety-like behavior, and this suggests heterogeneity of the phenotypes (Günther et al., 2019). EC18 is an HCN4 blocker and it is efficacious in reducing seizure susceptibility, as shown in mice (Kharouf et al., 2020b). Gabapentin reduces HCN4 current in mice (Tae et al., 2017). Glycogen synthase kinase-3β (GSK3β) inhibits seizures (Urbanska et al., 2019). The blockage of HCN channels *via* ZD7288 antagonism in the ventrobasal thalamus decreases thalamocortical neuronal firing and eliminates spontaneous absence seizures in GAERS, Wistar rats, and male Stargazer mice (David et al., 2018).

### HCN Channel Auxiliary Subunits and Epilepsy

Currently, there is no known direct link between mutations of HCN auxiliary subunits and epilepsy. However, some variants of unknown significance have been reported, which indicate that existing dysfunctions may produce more information in the future (DiFrancesco et al., 2019). TRIP8b is a type of voltage-dependent cation channel that is modulated by direct cAMP binding, and it interacts with the C-terminal and CNBD of HCN channels to control channel trafficking or gating (Han et al., 2020). TRIP8b promotes HCN channel expression and enhances Ih current (Lewis et al., 2009). It hinders channel opening by shifting activation to more negative potentials (Santoro et al., 2009). TRIP8b modulates the trafficking of the HCN channel to dendrites; however, the modulation of the adult presynaptic cortical HCN expression is independent of TRIP8b (Huang et al., 2012). It enhances the distal dendritic enrichment of HCN channels in CA1 pyramidal neurons (Han et al., 2020). In addition, it is crucial for the regulation of thalamocortical oscillations since it can decrease HCN channel expression in the thalamocortical relay and cortical pyramidal neurons, with a possible mechanism that is secondary to reduced cAMP signaling, as shown in *TRIP8b*-deficient mice (TRIP8b-/-) (Budde et al., 2005; Zobeiri et al., 2018). *TRIP8b*-knockout mice serve as an animal model for absence epilepsy (Heuermann et al., 2016). TRIP8b–HCN interaction is regulated by phosphorylation: consequently, loss of TRIP8b phosphorylation may affect HCN function during the development of temporal lobe epilepsy (Foote et al., 2019). The loss of HCN1 in distal dendrites minimizes the interaction between TRIP8b and HCN1 channels in animal models of temporal lobe epilepsy, which implies that TRIP8b interaction with HCN1 is important for appropriate HCN1 channel function in CA1 pyramidal neuron dendrites (Shin et al., 2008). *TRIP8b*-knockout animal model of absence seizures demonstrates decreased HCN channel expression and function in thalamic-projecting cortical layer 5b neurons and thalamic relay neurons (Heuermann et al., 2016). However, HCN function is preserved in inhibitory neurons of the reticular thalamic nucleus (Heuermann et al., 2016). *TRIP8b*-null mice with kainic acid-induced seizures show that presynaptic adult cortical HCN channel expression continually diminishes following seizure occurrence, but there is no affection of dendritic HCN channels (Huang et al., 2012). Therefore, the modulation of adult presynaptic cortical HCN expression is independent of TRIP8b (Huang et al., 2012). Altogether, these animal model studies suggest that the function of TRIP8b in the regulation of HCN channels is neuronal-specific.

Potassium voltage-gated channel subfamily E regulatory subunit-2 (KCNE2), also known as MinK-related protein (MiRP1), belongs to the family of single-helix transmembrane proteins (5 members) that play a major role in regulating HCN channels (Brandt et al., 2009). They stand as a beta-subunit of HCN channels (Yu et al., 2001; Qu et al., 2004). KCNE2 can increase the Ih current density of the HCN channels (Brandt et al., 2009). Deletion of the *KCNE2* gene decreases the Ih current density and reduces brain expression of HCN1 and HCN2 (but not HCN4), which is associated with increased excitability in the cortico-thalamo-cortical loop neurons (Ying et al., 2012). *KCNE2* deletion also increases input resistance and temporal summation, with consequent increased intrinsic excitability and enhanced burst firing (Ying et al., 2012). It has been shown that the number of KCNE2 subunits that form the complex with HCN channels may differ according to the HCN isoform and may depend on their concentration (Lussier et al., 2019). For example, one study revealed that only the C-terminal of KCNE2, but not other KCNE subunits, can interact with HCN4 channels (Decher et al., 2003).

Hyperpolarization-activated cyclic nucleotide-gated channel 1 can interact and form a complex with F-actin-binding filamin A (Gravante et al., 2004; Ramakrishnan et al., 2012). Filamin A interacts with HCN1 by binding to the C-terminal to slow down HCN1 channel kinetics and to induce channel clustering in certain parts of the cell membrane, which decreases channel expression and whole-cell conductance (Gravante et al., 2004). Filamin A modulates internalization of HCN1 channels, which is associated with redistribution of HCN1 channels on cell membranes, accumulation of channels in endosomes, and reduction of Ih current (Noam et al., 2014). The redistribution of the HCN1 channel intensifies the efficiency of channel control *via* modulating agents (Gravante et al., 2004). The deletion of filamin A in hippocampal neurons facilitates the expression of HCN1 (Noam et al., 2014). Caveolin-3 (Cav3) is another important accessory subunit. An increase in the number of caveolae can enhance the function of HCN channels in diabetic cytopathy (Dong et al., 2016). HCN4 interacts with caveolin-3 during cardiomyocyte development (Bosman et al., 2013). The P104L mutation on caveolin-3 impairs HCN4 function and causes reduction in cardiac pacemaker activity (Ye et al., 2008).

Tamalin, also known as GRP1-associated scaffold protein (GRASP), has many separate protein regions, including PSD-95, discs large, zona occludens 1 (PDZ)—domain, glycine-rich, alanine-rich, proline-rich, leucine zipper sequence, and C-terminal PDZ-binding motif (Kitano et al., 2002). Tamalin is involved in multimolecular protein assembly in neurons, and it forms complexes with postsynaptic receptors or scaffold proteins, such as group 1 mGluRs, synaptic scaffolding molecule (S-SCAM), PSD-95, and SAP90/PSD-95-associated proteins (SAPAPs) (Kimura et al., 2004). It enhances intracellular trafficking and cell surface expression of group 1 mGluRs (Kimura et al., 2004). It has been shown that Tamalin interacts with HCN2 at both the PDZ-binding motif and C-terminal of HCN2 (Kimura et al., 2004), although its role is unknown. It remains unclear whether distorted Tamalin-HCN2 interaction can alter the Ih current or HCN2 expression. S-SCAM is a synaptic protein comprising PDZ, guanylate kinase, and two tryptophan (WW) domains. S-SCAM interacts with C-terminal of HCN2 *via* CNBD (Kimura et al., 2004). *S-SCAM-*knockout mice developed well and were born alive, although they died within 24 h (Iida et al., 2007). Future studies should explore whether distorted Tamalin-HCN2 interaction can alter Ih current or HCN2 expression.

Mint2, also known as APBA2 (amyloid beta-precursor protein-binding family A member 2), is a synaptic adaptor protein that plays a major role in excitatory synaptic transmission. It binds to Munc-18 (a protein that is important for synaptic vesicle exocytosis) and CASK (a protein essential for targeting and localization of synaptic membrane proteins) (Butz et al., 1998). Mint2 has two PDZ domains and a phosphotyrosine domain, and it interacts with amyloid precursor and Munc-18 proteins (Lewis et al., 2010). The CNBD downstream sequence of HCN2 may interact with Munc-18-interacting domains of Mint2 (Kimura et al., 2004). The interaction between HCN2 and Mint2 is essential for Mint2-mediated escalation of the HCN2 protein cellular contents (Kimura et al., 2004). Mint1-knockout mice exhibit enhanced release probability of aminobutyric acid in hippocampal interneurons (Ho et al., 2003); however, it is unknown whether impaired Mint1-HCN interaction can affect Ih current. In Alzheimer's disease (AD), amyloid-β has been reported to have a potential link between epilepsy and dementia. Some fluctuating dementias are currently attributed to unrecognized interictal epileptiform discharges and subclinical seizures (Sen et al., 2020; Romoli et al., 2021). There is an augmented burden of Aβ pathology in the brain in AD cases with epilepsy (Romoli et al., 2021). It has been shown that targeting the amyloid precursor protein Mint2 protein–protein interaction with a peptide-based inhibitor can reduce amyloid-β formation in a neuronal model of AD (Bartling et al., 2021). In addition, the amyloid precursor protein-binding-deficient Mint2 variant demonstrates a substantial reduction of the amyloid-β levels (Bartling et al., 2021). Currently, it is recommended that antiseizure medications with mood-stabilizing functions (such as lamotrigine) be administered instead of acetylcholinesterase inhibitors for the old patients with epilepsy, including AD cases (Sen et al., 2020). Lamotrigine can activate the HCN channel (Mader et al., 2018) and increase Ih current in rat hippocampal slices, which reduces neuronal firing and dendritic excitability (Poolos et al., 2002). Based on these studies, we speculate that the heightened interaction between Mint2 and HCN channels can increase seizure susceptibility because Mint1-knockout mice exhibit enhanced release probability of aminobutyric acid which is protective against epilepsy, amyloid precursor protein-binding-deficient Mint2 variant shows a considerable reduction of the amyloid-β levels which might correlate with the reduction of the epilepsy burden in AD cases, and the fact that the enhancement of the Ih current *via* lamotrigine can reduce neuronal firing and dendritic excitability. Nevertheless, more studies are required to confirm this hypothesis.

Phosphatidylinositol 4,5-bisphosphate (PIP2) is another important regulator of the HCN channels. HCN2 can be regulated by blocking P1P2 which decelerates the hyperpolarizing shift in activation, and by antibody-induced depletion of PIP2 which causes further hyperpolarizing shift in activation (Pian et al., 2006). These shifts in activation can be partially reversed by magnesium ATP, although this may also be blocked by Wortmannin (PI kinase inhibitor) (Pian et al., 2006). There is further evidence regarding the role of P1P2 in enhancing the gating properties of HCN1 and HCN2 (Pian et al., 2007). It has been revealed that changes in pH can alter HCN2 gating properties; but are independent of cAMP concentration (Lewis et al., 2010). Orexin A is an arousal peptide that can regulate HCN channels. In layer V pyramidal neurons of mice prelimbic cortex, orexin A represses Ih currents, shifts the activation curve in the negative direction, and enhances excitability of pyramidal neurons, thereby contributing to arousal and cognition (Li et al., 2010). The effect of orexin A depends on potassium channels and non-selective cation channels (Yan et al., 2012). Orexin A (hypocretin-1) employs a postsynaptic excitatory action on prefrontal cortex neurons by inhibiting potassium currents *via* the activation of protein kinase C (PKC) and phospholipase C (PLC) signaling pathways (Xia et al., 2005). Orexin deficiency is related to narcolepsy according to the reviews (Mahoney et al., 2019; Nepovimova et al., 2019; Mignot et al., 2021). Therefore, high Ih current due to the lack of orexin can play a role in the pathogenesis of narcolepsy.

### HCN Channel Regulators and Epilepsy

Cyclic AMP-mediated neurotransmitters regulate HCN channels by enhancing their opening. cAMP is the chief endogenous positive modulator of Ih current (Simeone et al., 2005). The binding of cAMP to the CNBD speeds HCN channel activation at more depolarized potentials (Wang et al., 2002; Robinson and Siegelbaum, 2003; Ulens and Siegelbaum, 2003). A study of rat thalamocortical neurons showed that rat growth or maturity is associated with increased mRNA and protein expression levels of HCN1 and HCN2, but less of HCN4 that interacts with cAMP to modulate Ih activity (Kanyshkova et al., 2009). Besides, cAMP and cGMP inhibit the HCN3 current (Mistrík et al., 2005). HCN2 and HCN4 isoforms are the most sensitive to cAMP, followed by HCN1, but HCN3 is not sensitive (Santoro and Shah, 2020). The absence seizures, impaired visual learning, and altered non-rapid eye movement sleep properties are noticed in *HCN2* knock-in mouse model (HCN2EA) in which the binding of cAMP to HCN2 is eliminated by two amino acid substitutions (R591E and T592A) (Hammelmann et al., 2019). Impaired binding of cAMP to HCN channels promotes epileptogenesis in Genetic Absence Epilepsy Rat from Strasbourg (GAERS) *via* enhancement of the T-current-mediated calcium ion signaling (Kuisle et al., 2006). The binding of cAMP to HCN channels in GAERS is weakened in the acute phase, thereby promoting seizures, and the compensatory mechanisms to stabilize Ih current activity may cause cessation of spike-wave discharges in chronic epilepsy.

P38 mitogen-activated protein kinase (p38 MAPK) is a strong modulator of HCN1 biophysical properties. The inhibition of p38 MAPK reduces Ih current in rats' hippocampal pyramidal neurons, and its activation improves Ih current (Poolos et al., 2006). The inhibition of p38 MAPK in rat's hippocampal pyramidal neurons caused 25 mV hyperpolarization of Ih activation with consequent hyperpolarization of resting potential, increased input resistance, and enhanced temporal summation of excitatory inputs (Poolos et al., 2006). In contrast, the activation of p38 MAPK by anisomycin caused 11 mV depolarizing shift of Ih activation in conjunction with depolarization of resting potential, reduced input resistance, and decreased temporal summation (Poolos et al., 2006). The increased input resistance and temporal summation of excitatory inputs can be the possible mechanisms for epilepsy. Short Stature Homeobox 2 (Shox2) regulates HCN current in thalamocortical neurons (Yu et al., 2021). *Shox2*-knockout mice exhibit the reduced expression of HCN2, HCN4, and Cav3.1 channels, which implies that Shox2 is an important transcription factor for these channels (Yu et al., 2021). Also, *Shox2*-knockout mice are more vulnerable to pilocarpine-induced seizures (Yu et al., 2021). Casein kinase II (CK2) is an active serine or threonine protein kinase, a tetramer of two alpha- and two beta-subunits. The inhibition of CK2 by 4,5,6,7-tetrabromotriazole in acute epilepsy slice models resulted in increased expression of HCN1 channels, HCN3 channels, and voltage-independent calcium (Ca^2+^)-activated potassium (K^+^) channels (KCa2.2), also known as small-conductance Ca^2+^-activated K^+^ channels (SK2), thereby producing antiepileptic effects (Schulze et al., 2020). When HCN channel blocker ZD7288 was administered, pretreatment with 4,5,6,7-tetrabromotriazole salvaged the hyperpolarizing potential and spike frequency adaptation *via* the activity of the KCa2.2 (Schulze et al., 2020).

Glycosylation is a post-translational protein modification that affects intracellular processes, such as the folding and trafficking of most glycoproteins. It can modulate and influence the total number of HCN channels in the membrane and channel heteromerization (Noam et al., 2011). It has been shown in HEK293 cells that N-linked glycosylation is required for cell surface trafficking of HCN channels (Much et al., 2003). Seizure activity increases the amount of glycosylated HCN1 but not HCN2 channel molecules, and blocking of HCN1 glycosylation abolishes seizure evoked increase of heteromerization (Zha et al., 2008). The developmental seizures stimulate the formation of hippocampal HCN1/HCN2 heteromeric channels due to the augmented amount of HCN2 channels (mRNA and protein levels), compared to HCN1 channels (Brewster et al., 2005). Heteromeric HCN channels activate hippocampal hyperexcitability, leading to the development of epilepsy (Brewster et al., 2005). In particular, seizures increase HCN1/HCN2 heteromerization in the hippocampus (Zha et al., 2008).

Protein kinase C (PKC) is a family of serine–threonine kinases located in many cell types, and they play many roles in signal transduction processes. The activation of PKC reduces Ih currents, HCN1 surface expression, and HCN1 channel phosphorylation in hippocampal principal neurons (Williams et al., 2015). However, the inhibition of PKC could reverse the situation (Williams et al., 2015), which indicates that the PKC pathway may be a good target for modulating HCN1 expression and Ih current. Phosphatase calcineurin (CaN) and p38 MAPK are the phosphorylation pathways in epilepsy that contribute to the downregulation of HCN channel gating, resulting in neuronal hyperexcitability (Jung et al., 2010). Tyrosine phosphorylation modulates HCN channels *via* tyrosine kinase Src. The receptor-like protein tyrosine phosphatase-alpha (RPTP-alpha) considerably inhibits or abolishes HCN2 channel expression in HEK293 cells, which is associated with decreased tyrosine phosphorylation in the channel protein (Huang et al., 2008). Alteration of the phosphorylation of HCN1 and HCN2 channels plays a role in chronic epileptogenesis, as shown in rats' CA1 hippocampal tissue and human brain tissue (Concepcion et al., 2021). Several phosphosites for HCN1 and HCN2 have been mapped in chronic epilepsy cases and animal models of temporal lobe epilepsy (Concepcion et al., 2021). Phosphosites for HCN1 include T41, S56, G80, T99, S116, T472, Y513, S599, T646, S770, T771, S846, S847, S871, S872, and S873 (Concepcion et al., 2021). Phosphosites for HCN2 include S80, S81, T87, S97, S110, S132, S146, S771, S779, A785, S786, S793, Y795, A801, A853, S860, S866, S868, and G873 (Concepcion et al., 2021). The HCN1 channel phosphosites S791 and S791D were implicated in the pathogenesis of chronic epilepsy in rats (Concepcion et al., 2021). Importantly, TRIP8b–HCN interaction is regulated by phosphorylation; therefore, the loss of TRIP8b phosphorylation may affect HCN function during the development of epilepsy (Foote et al., 2019).

SUMOylation was recently reported as a modulator of the Ih current (Parker et al., 2019). Post-translational SUMOylation regulates ion channel interactions; therefore, its dysregulation causes nervous system disorders, such as epilepsy (Parker et al., 2019). SUMOylation increases the surface expression of HCN2 channel, thereby augmenting Ih current in the mouse brain (Parker et al., 2016). HCN2 has several SUMOylation sites, including K464, K484, K534, and K669 (Parker et al., 2016). The K464, K534, and K484 sites are located in the C-linker which is necessary for channel trafficking and cAMP gating, whereas K669 enhances surface expression of the HCN2 channel (Parker et al., 2016). The K464 and K484 sites are in the C-linker that connects the CNBD to the transmembrane region, whereas K534 is found within the CNBD. SUMOylation in the C-linker and/or the distal fragment can promote or hinder the binding of the TRIP8b. It has been shown, in a rat model of complete Freund's adjuvant-induced hindpaw inflammation, that the increased Ih current in the early stages of inflammation is influenced by increased HCN2 protein expression and post-translational SUMOylation (Forster et al., 2020).

### Acquired HCN Channelopathies and Epilepsy

Hyperpolarization-activated cyclic nucleotide-gated channels are also related to inflammation-associated epilepsy. It has been shown that the injection of lipopolysaccharide (inflammatory molecule) into rats will elicit a strong and long-lasting inflammatory response in hippocampal microglia (Frigerio et al., 2018). This is accompanied by the upregulation of Toll-like receptors (TLR4), reduction of expression and protein levels of dendritic cAMP-HCN1 channels, reduction of TRIP8b, decrease of HCN1 current, and alteration of rhythmic activities (Frigerio et al., 2018). The injured cerebral cortex can develop seizures due to changes in HCN channel expression which increases neuronal excitability (Paz et al., 2013).

Febrile infection-related epilepsy syndrome (FIRES) is a rare epileptic condition that is preceded by febrile illness about 24 h−2 weeks prior to the onset of refractory status epilepticus, and the condition is not limited by age (Kessi et al., 2020b). This condition has high mortality rates, most cases die from cardiorespiratory failure, most cases do not respond to anticonvulsants or immunotherapies, and the underlying mechanism is unknown (Kessi et al., 2020b). In the acute phase, most cases (61%) have a normal brain scan, but 25% of cases have diffuse cerebral edema plus defects in the temporal lobes, basal ganglia, thalami, and brainstem (Culleton et al., 2019). In the chronic phase, there is brain atrophy and mesial temporal sclerosis (Culleton et al., 2019). The mice models of FIRES, which were made using low-dose lipopolysaccharide injection and hyperthermia, revealed that lipopolysaccharides cause seizure-induced proinflammatory cytokine production, microglial activation, disruption of blood–brain barrier, and sparse ischemic lesions in the cerebral cortex (Koh et al., 2020). Microglial activation leads to the production of proinflammatory cytokines, such as interleukin-1 (IL-1), interleukin-6 (IL-6), and tumor necrosis factor-alpha (TNF-α) (Smith et al., 2012). Inflammatory cytokines that include IL-1, TNF-α, and IL-6 can result in gliosis, as demonstrated in the brain biopsy of seven FIRES cases (van Baalen et al., 2010). The ketogenic diet's antiseizure and anti-inflammatory activity may provide effective therapy for the acute phase of FIRES, and cannabidiol is effective for the chronic phase of FIRES (Kessi et al., 2020b). Phenobarbital is an effective therapy in the acute and chronic phases of FIRES, despite the minimal evidence (Kessi et al., 2020b). Phenobarbital can increase the Ih current in hippocampal pyramidal neurons (Jung et al., 2007). Anakinra (human interleukin-1 receptor antagonist), a drug with anti-inflammatory activity, has been reported to be effective in the acute phase of FIRES (Kenney-Jung et al., 2016; Dilena et al., 2019; Lai et al., 2020; L'Erario et al., 2021). The cerebrospinal fluid of FIRES cases demonstrated high levels of proinflammatory cytokines before treatment, but the levels were normalized after anakinra therapy (Kenney-Jung et al., 2016).

Similar to HCN channels, the transient receptor potential vanilloid-1 (TRPV1) channel can respond to noxious heat and oxidative stress (Caterina et al., 1997). Moreover, the activation of the mitochondrial TRPV1 channel leads to microglial migration which is essential for neuroinflammation (Miyake et al., 2015). Capsazepine, a blocker of the TRPV1 channel, inhibits HCN1-mediated currents in a non-use-dependent manner (Gill et al., 2004). The proposed mechanisms of cannabidiol action include its ability to desensitize TRPV1 channels (Franco et al., 2021). Its antiseizure activity *via* TRPV1 results from its ability to modulate calcium ions release and thus neuronal excitability (Franco et al., 2021). Therefore, there may be a relationship between cannabidiol and HCN channels. We hypothesize that the efficacy of cannabidiol in the chronic phase of FIRES may be due to its ability to modulate channels, including HCN and TRPV1.

Although there is currently no direct evidence to support the involvement of HCN and TRPV1 channelopathies in the pathogenesis of FIRES, we believe or propose that they contribute to the pathogenesis. The initial febrile illness observed in FIRES can alter the expression of HCN channels in astrocytes and microglia, which may result in progressive status epilepticus, and permanent acquired channelopathy in the brain and heart. Inflammation in astrocytes and microglia can reduce HCN1 and HCN2 expression, leading to the reduction of inhibitory effect and enhanced neuronal excitability. Our previous published review on FIRES showed that most cases die from cardiorespiratory failure or SUDEP (Kessi et al., 2020b). The rat pilocarpine model of epilepsy is an animal model of spontaneous induced recurrent seizures, and it revealed that there is a diminished expression of dendritic HCN channels during the acute phase of epilepsy, which is accompanied by the loss of hyperpolarization of voltage-dependent activation (Jung et al., 2007). The phenomenon progresses to the chronic phase which increases neuronal excitability and consequent epileptogenesis (Jung et al., 2007). Phenobarbital can suppress seizures and reverse the neuronal current changes but not the protein expression changes (Jung et al., 2007). This may explain why phenobarbital is not as efficient as cannabidiol in reducing seizures during the chronic phase of FIRES. The loss of Ih current and HCN1channel expression starts 1 h after status epilepticus and involves several steps, including dendritic HCN1 channel internalization, deferred loss of protein expression, and finally the downregulation of mRNA expression (Jung et al., 2011). Consequently, we propose that FIRES may be a neuroinflammatory condition that produces acquired channelopathies. The clinical animal models of FIRES can be used to further explore whether there is acquired HCN and TRPV1 channelopathies.

### Other HCN-Related Epileptic Disorders

Epilepsy can reduce the expression of HCN channels, leading to increased neuronal excitability in dorsal hippocampal CA1 neurons (Arnold et al., 2019). The disruption of Ih current disturbs hippocampal theta function in rat models of temporal lobe epilepsy (Marcelin et al., 2009), and during seizures, there is a reduced expression of HCN1 channel and upregulation of HCN2 channel (Richichi et al., 2008). According to one study, messenger RNA and protein expression of HCN1 and HCN2 were downregulated in controls compared to the cases with medial temporal lobe epilepsy and hippocampal sclerosis (Lin et al., 2020). In these cases of mesial temporal lobe epilepsy with hippocampal sclerosis, the lowered expression of HCN1 and HCN2 during chronic phases reduces Ih current density and function, thereby decreasing inhibitory effects and enhancing neuronal excitability (Lin et al., 2020). The downregulation of HCN1 expression after seizures augments dendritic excitability, resulting in the development of temporal lobe epilepsy (Powell et al., 2008). The mechanisms for HCN1 downregulation include calcium-permeable AMPA receptor-mediated calcium ion influx, followed by the activation of calcium or calmodulin-dependent protein kinase II (Richichi et al., 2008). The other proposed mechanism is the alteration of phosphorylation signaling that is facilitated by phosphatase calcineurin (CaN) or p38 MAPK (Jung et al., 2010). Another study showed that after status epilepticus, there is reduced expression of HCN channels and reduced relocalization from distal dendrites to soma, which influence CA1 excitability (Shin et al., 2008).

The animal model of Rett syndrome [Mecp2 (-/y)] CA1 exhibits heightened neuronal excitability as the pH increases from normal to high (pH 7.4–8.4) (Balakrishnan and Mironov, 2018). This alkaline pH enhances neuronal excitability *via* the loss of the Ih current and modulation of calcium channels (Balakrishnan and Mironov, 2018). HCN channels function as voltage absorbers and reduce dendritic membrane resistance in pyramidal neurons, resulting in reduced membrane excitability (Kase and Imoto, 2012). Consequently, modulation of pH and magnesium levels in Rett syndrome can modulate the expression and functions of HCN and calcium channels, which may reduce epilepsy (Balakrishnan and Mironov, 2018).

Hyperpolarization-activated cyclic nucleotide-gated channels play a role in epilepsy that is related to cortical development malformations. HCN channels modulate excitatory postsynaptic potentials in diverse classes of GABAergic interneurons *via* different ways, and this function is altered in malformed rat neocortex (Albertson et al., 2017). In rats' L5 pyramidal neurons with induced focal cortical dysplasia, there is a reduced Ih current in distal dendrites, resulting in increased intrinsic and synaptic excitability (Hablitz and Yang, 2010; Albertson et al., 2011). Induction of cortical dysplasia in rats during prenatal period reduces the expression of *HCN1* and *HCN2* genes in CA1 and CA3 pyramidal neurons, although none of the rats exhibit seizure activity (Işler et al., 2008).

### A General Overview of Treatments

Based on the literature, few cases achieved seizure freedom without receiving any antiseizure medication, less than half of the cases became seizure-free after receiving one or more antiseizure medications, including sodium valproate, and some developed drug-resistant epilepsy, even after receiving sodium valproate. It was shown that HCN1 M294L heterozygous knock-in (HCN1M294L) mouse demonstrates the clinical manifestations of patients with the *HCN1* M305L variant, including spontaneous seizures, seizure exaggeration by lamotrigine, and the seizure reduction by sodium valproate (Bleakley et al., 2021). Nevertheless, we attempted to do some statistics to see whether sodium valproate has any association with seizure freedom but there was no correlation (*p*-value = 0.717). Therefore, it is difficult to tell which antiseizure medication can result in seizure freedom; however, sodium valproate can be tried for cases with epilepsy of unknown origin and genetically positive screening for HCN channelopathy.

Some antiseizure medications work through or act *via* HCN channels as shown on cell culture models, animal models, and human brain slices. Lamotrigine can activate HCN channel (Mader et al., 2018) and increase Ih current in rat hippocampal slices, which reduces neuronal firing and dendritic excitability (Poolos et al., 2002). Through the whole-cell and cell-attached recordings, it has been shown in rat hippocampal slices that lamotrigine selectively reduces action potential firing from dendritic depolarization, whereas it minimally affects firing at the soma, suggesting that lamotrigine targets dendrites specifically (Poolos et al., 2002). In addition, there is the evidence from human cortical pyramidal neurons that lamotrigine reduces input resistance and enhances Ih current in layers 2/3 in patients with drug-resistant epilepsy (Lehnhoff et al., 2019). Based on the electrophysiological studies on Xenopus oocytes, mouse spinal cord slices targeting either parvalbumin-positive or calretinin-positive inhibitory neurons, it was revealed that there is a hyperpolarizing shift in the V1/2 of Ih current measured from HCN4-expressing PV+ inhibitory neurons in the spinal dorsal horn but not calretinin-positive inhibitory neurons (Tae et al., 2017). In contrast, another study of CA1 pyramidal cells in hippocampal slices has shown that gabapentin can increase Ih current *via* cAMP-independent mechanism (Surges et al., 2003). Phenobarbital can increase Ih current in rat pilocarpine model of epilepsy but cannot reverse the loss of HCN channel expression in CA1 hippocampal pyramidal neurons (Jung et al., 2007). Acetazolamide can enhance Ih current in rat thalamic relay neurons *via* intracellular alkalinization (Munsch and Pape, 1999). Ethosuximide can suppress seizures in HCN1-knockout rats whose cortical and hippocampal pyramidal neurons exhibited a significant reduction of Ih current, although its mechanism of action is not clear (Nishitani et al., 2019). We speculate that ethosuximide can increase Ih current in cortical and hippocampal pyramidal neurons. Capsazepine, a blocker of the TRPV1 channel, inhibits HCN1-mediated currents in a non-use-dependent manner as shown in CV-1 and CHO cell lines (Gill et al., 2004).

Notably, some of the anesthetics used in status epilepticus work through HCN channels too. Propofol can inhibit HCN1 subunit mediated Ih current in mouse cortical pyramidal neurons (Chen et al., 2005). Ketamine has been shown to be effective and relatively safe for the control of multidrug-resistant refractory status epilepticus in children and adults (Fang and Wang, 2015). In cortical pyramidal neurons, ketamine was suggested to work *via* HCN1 channels (Chen et al., 2009a). Likewise, isoflurane has been reported to associate with a good effect in stopping refractory status epilepticus or super refractory status epilepticus (Stetefeld et al., 2021). Isoflurane can affect neuronal Ih current as shown in cortical pyramidal neurons of HCN1-knockout mice (Chen et al., 2009b).

## Conclusion

Hyperpolarization-activated cyclic nucleotide-gated channels are widespread in the body especially in the brain, microglia, and astrocytes. HCN channel auxiliary subunits include TRIP8b, KCNE2, actin-binding protein filamin A, caveolin-3, Tamalin, S-SCAM, and Mint2. HCN channels can be regulated *via* cAMP system, p38 MAPK, Shox2, CK2, N-linked glycosylation, PKC, phosphorylation, and SUMOylation. HCN channelopathies are implicated in epilepsy. Pathogenic variants of *HCN1, HCN2, HCN3*, and *HCN4* have been reported to be associated with epilepsy in 74 cases, and they have diverse phenotypes. Less than half of the cases with HCN channelopathies achieve seizure-freedom. A total of twelve cases (16.2%) died due to SUDEP: *HCN1* (*n* = 4), *HCN2* (*n* = 2), *HCN3* (*n* = 2), and *HCN4* (*n* = 4).

For the *HCN1* gene, majority of the cases can present with either febrile seizures, or febrile seizure plus or genetic generalized epilepsy with febrile seizure plus or genetic/idiopathic generalized epilepsy. Some of the variants are related to different epileptic syndromes, which suggest heterogeneity of the *HCN1* phenotypes. Besides epilepsy, intellectual disability, behavioral disturbances, autistic features, polyphagia, motor delay, ADHD, truncal ataxia, language delay, and microcephaly can be noticed in the cases with *HCN1* pathogenic variants. According to the electrophysiological studies on cell culture models, both GOF and LOF *HCN1* variants are associated with epilepsy, but LOF is associated with more severe phenotypes, including 3 deaths due to the deletion and a strong reduction in HCN1 current density. The underlying mechanisms of epilepsy for both GOF and LOF variants remain unclear since most of the functional studies were limited to the electrophysiological studies and only a few were done to explore the neuroanatomical localization in different neuronal subtypes not to mention the distribution patterns. Most rodent models of epilepsy (especially absence seizures) support the fact that the loss of the HCN1 current is associated with the occurrence of epilepsy in pyramidal, cortical, and thalamic neurons, but there is limited evidence showing that the upregulation of this current in the same or other neurons can produce epilepsy.

Most of the cases carrying *HCN2* gene pathogenic variants can present clinically with either febrile seizures, or febrile seizure plus or genetic generalized epilepsy with febrile seizure plus or genetic or idiopathic generalized epilepsy. Similar to *HCN1* gene, some of the variants are related to different epileptic syndromes which suggest heterogeneity of the *HCN2* phenotypes. Based on the electrophysiological studies on cell models, both GOF and LOF *HCN2* variants are related to epilepsy. However, GOF effect is more common in *HCN2* gene. The proposed mechanisms of epilepsy for the GOF variants include continuous cation leak, N-terminal deletion, increased HCN1 expression and current, suppression of HCN2 and HCN4 currents, reduction of cAMP sensitivity, reduction of the GABAA receptor-mediated synaptic inhibition, depolarizing shift in the resting membrane potential, impaired input resistance, and cAMP-independent enhanced availability of Ih during high temperatures, whereas the suggested mechanism of epilepsy for the LOF variants includes the loss of the voltage dependence in activation and deactivation leading to continuous excitatory cation flow. Besides, the loss of HCN2 channels on interneurons and thalamocortical relay neurons has been suggested as a possible underlying mechanism of epilepsy as shown in simulation study on a hippocampal CA1 pyramidal neuron synapse model and HCN2-null mouse. For the *HCN4* gene, most cases present with genetic or idiopathic generalized epilepsy. Most cases had LOF variants. Evidence from the animal models suggests that HCN4 channels are the controllers of brain excitability, and their inhibition reduces thalamocortical bursting firing. Nevertheless, it is unclear whether the same protective activity against epilepsy can be observed in other types of neurons. Currently, there is no direct link between mutations of HCN auxiliary and regulatory subunits with epilepsy in humans. However, there are some variants of unknown significance that have been reported, which indicate possibilities of future occurrences or research. Therefore, future studies should explore links between these unknown variants and epilepsy. HCN channels are also involved in the epileptogenesis of temporal lobe epilepsy, Rett syndrome, malformation of cortical development, and possibly FIRES. Since HCN channels are also expressed in astrocytes and microglia, future studies should explore their relation with inflammatory-related epilepsy, including FIRES.

The regulation of neuronal excitability *via* HCN channels depends on their neuroanatomical locations in the brain as well as neuronal distribution patterns: dendrites, axons, and soma of specific neurons. Although there are several rodent animal models, most of them focused to study the absence seizures. There are few animal models focused to explore the specific neuroanatomical location and distribution patterns of the reported variants, thus hampering to know more about the mechanisms of both GOF and LOF variants in causing epilepsy. Besides pharmacological targets and modulators, precise antiseizure medications are yet to be found. Currently, there are some HCN1 channel blockers with low efficiency. New HCN1-blocking drugs with high affinity should be developed. Since HCN1 current seems to be essential for the prevention of certain types of epilepsy, there is also the need to develop channel openers. There is no HCN2 channel opener, and this should be developed in the future. Due to the fact that HCN3 channelopathy is related to epilepsy and death, future studies may explore its association with the occurrence of epilepsy in living patients. HCN4 channels are the controllers of brain excitability, and blockers of these channels can be effective antiseizure medications. Since the reported HCN4 cases had epilepsy and carried pathogenic variants with LOF effects, there is a need to investigate how LOF mutations produce seizures whereas the brain-specific *HCN4*-knockout adult mice exhibit reduced seizure susceptibility.

Interestingly, the blockage of HCN1-mediated Ih current in the pyramidal, cortical, and thalamic neurons contributes to epilepsy, whereas blockers of HCN3- and HCN4-mediated currents suppress seizures. Likewise, propofol can block HCN2 current, leading to the reduction of neuronal excitability and burst firing in thalamocortical neurons. This finding suggests that HCN isoform-selective blockage might have different effects on seizure susceptibility (Kharouf et al., 2020b). This phenomenon has been hypothesized to occur due to the fact that HCN channel isoforms might have different biophysical features, anatomical expression, and functions in regulating neuronal excitability. For instance, in thalamocortical neurons, HCN4 channels are highly found in the soma; in contrast, HCN2 channels are highly expressed in dendritic spines (Abbas et al., 2006). Consequently, there is a need to develop HCN isoform-selective compounds that are based on biophysical features, anatomical expression, and functions.

## Recommendations

The availability of few cases of HCN-related epilepsy in the literature may suggest that less attention is being paid to the affected cases. We recommend that clinicians should include *HCN* genes in epilepsy gene panels. Researchers should do further genetic screening and consider HCN channel roles in epileptic syndromes of unknown cause and/or mechanisms with clues of possible neuroinflammation (including FIRES). Future studies should try to explore the possible underlying mechanisms for both GOF and LOF variants in the occurrence of epilepsy by focusing on exploring the specific neuronal subtypes, neuroanatomical location, and distribution patterns of each identified pathogenic variant. Future research should identify specific HCN channel openers and blockers with high binding affinity, which may help to produce targeted treatments.

## Data Availability Statement

The original contributions presented in the study are included in the article/Supplementary Material, further inquiries can be directed to the corresponding author.

## Author Contributions

MK designed the study, reviewed the articles, drafted, and wrote the manuscript. OB, HD, HH, BC, JX, and KK assisted in the literature review and preparation of tables and figures. OB, JP, YW, LY, GW, and FY revised the manuscript and supervised each step involved in the preparation of the manuscript. All authors have read and agreed to the content of the manuscript.

## Funding

This work was supported by the National Natural Science Foundation of China (No. 81771408), the National Natural Science Foundation of China (No. 81801297), and the Hunan Key Research and Development Program (No. 2019SK2081).

## Conflict of Interest

The authors declare that the research was conducted in the absence of any commercial or financial relationships that could be construed as a potential conflict of interest.

## Publisher's Note

All claims expressed in this article are solely those of the authors and do not necessarily represent those of their affiliated organizations, or those of the publisher, the editors and the reviewers. Any product that may be evaluated in this article, or claim that may be made by its manufacturer, is not guaranteed or endorsed by the publisher.
