# Supplementary material for: The Contribution of HCN Channelopathies in Different Epileptic Syndromes, Mechanisms, Modulators, and Potential Treatment Targets: A Systematic Review
Source: Front Mol Neurosci. 2022 May 19;15:807202. doi: 10.3389/fnmol.2022.807202 (PMC9161305; doi:10.3389/fnmol.2022.807202)
Supplement: Supplementary file 1 [file Table_1.docx]

**Supplementary Table 1: HCN channel genes reported to associate with epilepsy; genotypes and phenotypes**

| **Names of the gene** | **Age at seizure onset/sex** | **Initial seizures** | **Seizure types during disease course** | **Syndrome/**  **Phenotype according to primary author** | **Presence of status epilepticus** | **Epilepsy syndromes re-classification** | **Other clinical features/ organs affected** | **Inheritance/nucleotide or protein change/location** | **Altered protein function according to the electrophysiological studies/ClinVar** | **Silico modeling prediction/ACMG classification** | **MRI results** | **EEG finding** | **Therapies used** | **Prognosis** | **Reference** |
| --- | --- | --- | --- | --- | --- | --- | --- | --- | --- | --- | --- | --- | --- | --- | --- |
| *HCN1* | 10m/F | FS | FS, TCS, CS, absences, and focal, myoclonic seizures | Fever-sensitive EIEE | Yes | Fever-sensitive EIEE | Moderate ID and autistic features | Heterozygous de novo c.299C>T (p.S100F) | GOF (dominant negative effects) | P | Normal | \| GSW \| \| --- \| | \| VPA, CLB \| \| --- \| | Drug resistant EP | (Nava et al., 2014) |
| *HCN1* | 4m/F | FS and TCS | FS, TCS, absence, focal, and myoclonic seizures | Fever-sensitive EIEE | Yes | Fever-sensitive EIEE | Moderate to severe ID, behavioral disturbances, autistic features and polyphagia | Heterozygous de novo c.1201G>C (p.D401H) | GOF | P | Normal | PSW | LTG, CLB, VPA, PB | Drug resistant EP | (Nava et al., 2014) |
| *HCN1* | 8m/ F | FS and hemiclonic | FS, CS, focal, and absence | Fever-sensitive EIEE | Yes | Fever-sensitive EIEE | Severe ID, abnormal behavior, autistic features and motor delay | De novo, c.814T>C (p.S272P) | LOF (dominant-negative effects) | P | Normal | Normal then multifocal spikes, slowing | ETX, LTG | Drug resistant EP | (Nava et al., 2014) |
| *HCN1* | 8m/F | FS and hemiclonic | FS, focal, and absence | Fever-sensitive EIEE | Yes | Fever-sensitive EIEE | Moderate to severe ID, behavioral disturbances, and autistic features | De novo, c.890G>C (p.R297T) | LOF (dominant-negative effects) | P | Normal | Synchronous spike and wave with activation during sleep | VPA, CLB, STP, TPM, ZNS, LVT, LTG,  ketogenic diet, VNS | Drug resistant EP | (Nava et al., 2014) |
| *HCN1* | 13m/M | Atypical FS | FS (atypical), CS,  TCS, and absence | Fever-sensitive EIEE | No | Fever-sensitive EIEE | Mild ID, behavioral disturbances, ADHD and truncal ataxia | De novo c.835C>T (p.H279Y) | GOF | P | Normal | Multifocal epileptic discharges with fronto-central maximum | VPA, CLB, TPM | Drug resistant EP | (Nava et al., 2014) |
| **Names of the gene** | **Age at seizure onset/sex** | **Initial seizures** | **Seizure types during disease course** | **Syndrome/**  **Phenotype according to primary author** | **Presence of status epilepticus** | **Epilepsy syndromes re-classification** | **Other clinical features/ organs affected** | **Inheritance/nucleotide or protein change/location** | **Altered protein function according to the electrophysiological studies/ClinVar** | **Silico modeling prediction/ACMG classification** | **MRI results** | **EEG finding** | **Therapies used** | **Prognosis** | **Reference** |
| *HCN1* | 2y/M | FS | TCS | Genetic generalized epilepsy | No | Idiopathic/genetic generalized epilepsy | None | De novo heterozygous c.469C > G (p.L157V) | LOF (dominant-negative effects) | P | Normal | Generalized sharp-waves, prevalent on the bilateral anterior regions | VPA | Controlled seizure | (Bonzanni et al., 2018) |
| *HCN1* | M | Unknown | Unknown | Generalized epilepsy | No | Idiopathic/genetic generalized epilepsy | NIL | Familial Heterozygous c.469C > G (p.L157V) | LOF (dominant-negative effects) | LP | Unknown | Unknown | Unknown | Unknown | (DiFrancesco et al., 2019) |
| *HCN1* | 72 m/M | Unknown | Unknown | Childhood absence epilepsy | No | Idiopathic/genetic generalized epilepsy | NIL | De novo c.1769G>A  (p.R590Q) | LOF | LP | Unknown | Unknown | Unknown | Unknown | (DiFrancesco et al., 2019) |
| *HCN1* | 12 m/F | TCS | TCS, and atonic seizures | EIEE | Not clear | EIEE | Severe ID | De novo c.428T>A  (p.F143Y) | LOF | LP | Normal | Multifocal discharges | TPM, LTG, VPA, CBZ, CLB | Daily | (Marini et al., 2018) |
| *HCN1* | 5 m/F | FS | Focal and TCS | EIEE | Not clear | EIEE | Fever sensitive seizures, mild ID | De novo c.459G>C  (p.M153I) | GOF (faster activation) | P | Normal | Multifocal fronto-centro-temporal spikes | VPA, TPM, CBZ, CLB, LCM, STP; VPA+STP+CLB | Daily | (Marini et al., 2018) |
| *HCN1* | 9 m/M | TCS | TCS and focal seizures | Unclassified epilepsy infantile  onset | Not clear | Not clear | Mild language delay | De novo c.459G>C  (p.M153I) | GOF | P | Normal | Bifrontal sharply contoured rhythmic theta | LEV, CLB, OXC, ZNS | Weekly | (Marini et al., 2018) |
| **Names of the gene** | **Age at seizure onset/sex** | **Initial seizures** | **Seizure types during disease course** | **Syndrome/**  **Phenotype according to primary author** | **Presence of status epilepticus** | **Epilepsy syndromes re-classification** | **Other clinical features/ organs affected** | **Inheritance/nucleotide or protein change/location** | **Altered protein function according to the electrophysiological studies/ClinVar** | **Silico modeling prediction/ACMG classification** | **MRI results** | **EEG finding** | **Therapies used** | **Prognosis** | **Reference** |
| *HCN1* | 7 m/F | FS | FS, afebrile TCS, TS and absence | Generalized epilepsy | Not clear | Idiopathic/genetic generalized epilepsy | Fever sensitive seizures, mild ID and autistic features | De novo c.514A > C (p.T172P) | Unknown | P | Normal | Slow background | VPA, CLB, PB | Seizure free | (Marini et al., 2018) |
| *HCN1* | 13 m/F | FS | FS, afebrile TCS, absence | Febrile seizure plus | Not clear | Atypical FS | Fever sensitive seizures, moderate ID | De novo c.728T>G (p.M243R) | LOF | P | Normal | Normal | VPA, LTG | Rare seizures | (Marini et al., 2018) |
| *HCN1* | 10 m/F | FS, and TCS | FS | Febrile seizures | Not clear | FS | Fever sensitive seizures | De novo c.728T>G (p.M243R) | LOF | P | Normal | Normal | VPA | Seizures when have fever | (Marini et al., 2018) |
| *HCN1* | 8 m/F | FS and CS | Clonic generalized | Febrile seizure plus | Not clear | Atypical FS | Fever sensitive seizures | De novo c.779C > T (p.T260I) | Unknown | P | Normal | Normal | CLB when fever | Monthly | (Marini et al., 2018) |
| *HCN1* | 9 m/M | FS and CS | TCS | Unclassified epilepsy infantile | Not clear | Not clear | Mild language  delay | De novo c.790A > T  (p. S264C) | Unknown | P | Normal | Unknown | VPA, CBZ | Rare | (Marini et al., 2018) |
| *HCN1* | 42 m/F | Focal | Focal, TCS and myoclonic seizures | Childhood focal epilepsy | Not clear | Not clear | Moderate ID | De novo c.824T > C  (p.I275T) | Unknown | P | Thin corpus callosum | Unknown | VPA,LCM; LTG, TPM, KD | Daily | (Marini et al., 2018) |
| *HCN1* |  | TCS | TCS | Unclassified epilepsy infantile | Not clear | Not clear | Febrile sensitive seizures and mild DD | De novo c.913A>T (p.M305L) | No current recorded | P | Normal | Normal | GVG, LEV, PHT, PB, CLN, TPM, VPA. | Seizure free | (Marini et al., 2018) |
| **Names of the gene** | **Age at seizure onset/sex** | **Initial seizures** | **Seizure types during disease course** | **Syndrome/**  **Phenotype according to primary author** | **Presence of status epilepticus** | **Epilepsy syndromes re-classification** | **Other clinical features/ organs affected** | **Inheritance/nucleotide or protein change/location** | **Altered protein function according to the electrophysiological studies/ClinVar** | **Silico modeling prediction/ACMG classification** | **MRI results** | **EEG finding** | **Therapies used** | **Prognosis** | **Reference** |
| *HCN1* | 2 m/F | TCS | TCS, TS, focal, and CS | EIEE | Not clear | EIEE | Febrile sensitive seizures, severe ID, and microcephaly | De novo c.913A>T  (p.M305L)  S5 domain | No current recorded | P | Small calvarium, large cerebellum and incomplete myelination | Diffuse slowing of the background | STP, VPA, TPM, KD | Seizure free | (Marini et al., 2018) |
| *HCN1* | 30 h/M | TCS | TCS | Neonatal-onset epileptic encephalopathy (MMPSI) | Not clear | EIEE | Severe ID, microcephaly | De novo c.1171G>T  (p.G391D) | LOF | P | Mild, diffuse white matter hyper intensity | Multifocal and migrating focal seizures | PB, PHT, VPA, ACTH, RFN, ketamine, propofol, thiopental, CLN, midazolam, CLB, GVG, LEV, parampanel, lorazepam, CBZ, TPM | Died at 14 m due to cardiopulmonary failure | (Marini et al., 2018) |
| *HCN1* | 48 h/M | TCS | TCS with cyanosis | Neonatal-onset epileptic encephalopathy | Not clear | EIEE | Severe ID | De novo c.1171G>T (p.G391D) | LOF | P | Severe atrophy mainly fronto temporal. | Multifocal discharges | PB, LEV, GVG, CBZ, VPA, pyridoxine, TPM, KD, midazolam, thiopental, LTG, PHT, | Died at 15 m due to cardiopulmonary failure | (Marini et al., 2018) |
| **Names of the gene** | **Age at seizure onset/sex** | **Initial seizures** | **Seizure types during disease course** | **Syndrome/**  **Phenotype according to primary author** | **Presence of status epilepticus** | **Epilepsy syndromes re-classification** | **Other clinical features/ organs affected** | **Inheritance/nucleotide or protein change/location** | **Altered protein function according to the electrophysiological studies/ClinVar** | **Silico modeling prediction/ACMG classification** | **MRI results** | **EEG finding** | **Therapies used** | **Prognosis** | **Reference** |
| *HCN1* | 5 m/F | FS | FS; afebrile TCS, focal hypotonia, associated with cyanosis and vomit | FS plus | Not clear | Atypical FS | Fever sensitive seizures | De novo c.1171G>T (p.G391S) | GOF | P | Unknown | Normal background; no PA | VPA | Yearly | (Marini et al., 2018) |
| *HCN1* | 7 m/M | Hemiclonic | Focal and TCS | Genetic epilepsy with febrile seizures plus | Not clear | Idiopathic/genetic generalized epilepsy | Mild ID | De novo c.1171G>T (p.G391S) | GOF | P | Normal | Bilateral occipital PA | LEV | Yearly | (Marini et al., 2018) |
| *HCN1* | Infancy/F | Eyelid myoclonic | TCS, focal, absence and myoclonic | Generalized epilepsy with eyelid myoclonus | Not clear | Idiopathic/genetic generalized epilepsy | Moderate ID, and autistic traits | De novo c.1172G>A (p.G391C) | LOF | P | Normal | Unknown | Unknown drugs | Monthly | (Marini et al., 2018) |
| *HCN1* | 5 m/M | Hypotonia and cyanosis few h after vaccination | Myoclonic, focal and TCS | EIEE | Not clear | EIEE | Severe ID, autistic  traits | De novo c.1189A>C (p.I397L) | Nor clear | P | Normal | Bifrontal discharges at onset, later both focal and generalized epileptiform discharges | Primidone, TPM, VPA, LTG, CLN, sultiame, PHT, LEV, pregabalin | Daily | (Marini et al., 2018) |
| *HCN1* | 4 m/M | FS | TCS with apnea | EIEE | Not clear | EIEE | Febrile sensitive seizures, and severe ID | De novo c.1195T>C  (p.S399P) | LOF | P | Normal | multifocal and generalized spike waves during sleep | PB, LEV, CLN currently VPA & TPM | Daily | (Marini et al., 2018) |
| **Names of the gene** | **Age at seizure onset/sex** | **Initial seizures** | **Seizure types during disease course** | **Syndrome/**  **Phenotype according to primary author** | **Presence of status epilepticus** | **Epilepsy syndromes re-classification** | **Other clinical features/ organs affected** | **Inheritance/nucleotide or protein change/location** | **Altered protein function according to the electrophysiological studies/ClinVar** | **Silico modeling prediction/ACMG classification** | **MRI results** | **EEG finding** | **Therapies used** | **Prognosis** | **Reference** |
| *HCN1* | 72 m/M | Absence | Absence and TCS | Childhood absence epilepsy | Not clear | Idiopathic/genetic generalized epilepsy | None | De novo c.1769G>A(  p.R590Q) | LOF | LP | Normal | 3 Hz GSW | VPA + ESM, LEV; ZNS; LTG; TPM | Daily | (Marini et al., 2018) |
| *HCN1* | 8 m/M | FS | FS and afebrile TCS | Genetic epilepsy with febrile seizures plus | Not clear | Idiopathic/genetic generalized epilepsy | Unknown | Inherited c.512C>G  (p.T171R) | Unknown | P | Unknown | Unknown | PB, currently no AEDs | Controlled seizures | (Marini et al., 2018) |
| *HCN1* | 8 m/M | FS | FS and TCS | Genetic epilepsy with febrile seizures plus | Not clear | Idiopathic/genetic generalized epilepsy | Borderline ID | Inherited c.512C>G (p.T171R) | Unknown | P | Normal | Unknown | PB + GVG + VPA currently no AEDs | Seizure free | (Marini et al., 2018) |
| *HCN1* | 10 m/F | FS | FS | Genetic epilepsy with febrile seizures plus | Not clear | Idiopathic/genetic generalized epilepsy | Mild ID | Inherited c.512C>G  (p.T171R) | Unknown | P | Normal | Unknown | VPA, ESM, currently  no AEDs | Seizure free | (Marini et al., 2018) |
| *HCN1* | 11 m/F | FS | FS | Genetic epilepsy with febrile seizures plus | Not clear | Idiopathic/genetic generalized epilepsy | Mild ID | Inherited c.512C>G  (p.T171R) | Unknown | P | Normal | Unknown | VPA | Seizure free | (Marini et al., 2018) |
| *HCN1* | 3 m/F | FS | FS, febrile, and afebrile TCS | Genetic epilepsy with febrile seizures plus | Not clear | Idiopathic/genetic generalized epilepsy | None | Inherited c.986G>C  (p.C329S) | LOF | LP | Unknown | Unknown | CBZ | Seizure free | (Marini et al., 2018) |
| *HCN1* | 1 y/F | FS | FS | Febrile seizures | Not clear | FS | None | Inherited c.986G>C  (p.C329S) | LOF | LP | Unknown | Unknown | VPA | Seizure free | (Marini et al., 2018) |
| **Names of the gene** | **Age at seizure onset/sex** | **Initial seizures** | **Seizure types during disease course** | **Syndrome/**  **Phenotype according to primary author** | **Presence of status epilepticus** | **Epilepsy syndromes re-classification** | **Other clinical features/ organs affected** | **Inheritance/nucleotide or protein change/location** | **Altered protein function according to the electrophysiological studies/ClinVar** | **Silico modeling prediction/ACMG classification** | **MRI results** | **EEG finding** | **Therapies used** | **Prognosis** | **Reference** |
| *HCN1* | 18 m/F | FS | TCS without fever | Generalized epilepsy | Yes | Idiopathic/genetic generalized epilepsy | None | Inherited c.986G>C  (p.C329S) | LOF | LP | Normal | Unknown | VPA | Seizure free | (Marini et al., 2018) |
| *HCN1* | 8 m/M | FS and TCS | Febrile TCS | Generalized epilepsy | Not clear | Idiopathic/genetic generalized epilepsy | Mild ID | Inherited c.986G>C  (p.C329S) | LOF | LP | Normal | Unknown | VPA | Seizure free | (Marini et al., 2018) |
| *HCN1* | 13 m/F | FS | FS | Genetic epilepsy with febrile seizures plus | Not clear | Idiopathic/genetic generalized epilepsy | None | Inherited c.986G>C (p.C329S) | LOF | LP | Normal | Unknown | CBZ | Seizure free | (Marini et al., 2018) |
| *HCN1* | 18 m/F | FS | FS, febrile and  afebrile TCS | Genetic epilepsy with febrile seizures plus | Not clear | Idiopathic/genetic generalized epilepsy | Borderline ID | Inherited c.1240G>A  (p.V414M) | GOF | LP | Normal | Unknown | VPA | Seizure free | (Marini et al., 2018) |
| *HCN1* | 18 m/F | FS | FS | Genetic epilepsy with febrile seizures plus | Not clear | Idiopathic/genetic generalized epilepsy | None | Inherited c.1240G>A  (p.V414M) | GOF | LP | Normal | Unknown | No AEDs | Seizure free | (Marini et al., 2018) |
| *HCN1* | 12 m/M | FS | FS | Febrile seizure | Not clear | FS | None | Inherited c.1240G>A  (p.V414M) | GOF | LP | Unknown | Unknown | No AEDs | Seizure free | (Marini et al., 2018) |
| *HCN1* | 7 y/F | TCS | TCS | Generalized epilepsy | Not clear | Idiopathic/genetic generalized epilepsy | None | Inherited c.2039C>A (p.S680Y) | Unknown | LP | Normal | Unknown | VPA | Seizure free | (Marini et al., 2018) |
| **Names of the gene** | **Age at seizure onset/sex** | **Initial seizures** | **Seizure types during disease course** | **Syndrome/**  **Phenotype according to primary author** | **Presence of status epilepticus** | **Epilepsy syndromes re-classification** | **Other clinical features/ organs affected** | **Inheritance/nucleotide or protein change/location** | **Altered protein function according to the electrophysiological studies/ClinVar** | **Silico modeling prediction/ACMG classification** | **MRI results** | **EEG finding** | **Therapies used** | **Prognosis** | **Reference** |
| *HCN1* | Childhood/F | Absence | Absence | Childhood absence epilepsy | Not clear | Idiopathic/genetic generalized epilepsy | None | Inherited c.2039C>A (p.S680Y) | Unknown | LP | Normal | Unknown | No AEDs | Seizure free | (Marini et al., 2018) |
| *HCN1* | Mean age 40 y | Unknown | Unknown | Epilepsy plus SUDEP | Unknown | Not clear | Unknown | Unknown  Novel  G46V | Unknown | Nonsynonymous | Unknown | Unknown | Unknown | Died due to SUDEP | (Tu et al., 2011) |
| *HCN1* | 23y/F | Unknown | Unknown | Generalized epilepsy plus SUDEP | Unknown | Idiopathic/genetic generalized epilepsy | Unknown | p.72_74del (c.187_195delGGCGGTGGC) | Unknown | Variant of unknown significance | Unknown | Unknown | Unknown | Died due to SUDEP | (Coll et al., 2016) |
| *HCN2* | Mean age 40 y | Unknown | Unknown | Epilepsy plus SUDEP | Unknown | Not clear | Unknown | Unknown  Novel  F738C | Unknown | Nonsynonymous | Unknown | Unknown | Unknown | Died due to SUDEP | (Tu et al., 2011) |
| *HCN2* | Mean age 40 y | Unknown | Unknown | Epilepsy plus SUDEP | Unknown | Not clear | Unknown | Unknown  Novel  P802S | Unknown | Nonsynonymous | Unknown | Unknown | Unknown | Died due to SUDEP | (Tu et al., 2011) |
| *HCN2* | M | Unknown | Unknown | Generalized epilepsy | Not clear | Idiopathic/genetic generalized epilepsy | Not clear | Sporadic Heterozygous c.1543 G > A (p.E515K) | LOF | P | Not clear | Unknown | Not clear | Not clear | (DiFrancesco et al., 2019) |
| *HCN2* | 12y | Absence and TCS | Absence and TCS | Idiopathic Generalized Epilepsy | No | Idiopathic/genetic generalized epilepsy | None | Sporadic Heterozygous c.1543 G > A (p.E515K) | LOF | P | Normal | Generalized sharp-wave complexes | VPA, LTG, TPM, LEV | Drug resistant EP | (DiFrancesco et al., 2011) |
| **Names of the gene** | **Age at seizure onset/sex** | **Initial seizures** | **Seizure types during disease course** | **Syndrome/**  **phenotype** | **Presence of status epilepticus** | **Epilepsy syndromes re-classification** | **Other clinical features/ organs affected** | **Inheritance/nucleotide or protein change/location** | **Altered protein function** | **Silico modeling prediction/ACMG classification** | **MRI results** | **EEG finding** | **Therapies used** | **Prognosis** | **Reference** |
| *HCN2* | M | Focal seizures | Focal seizures | Idiopathic photosensitive occipital epilepsy | Not clear | Idiopathic/genetic generalized epilepsy | None | Inherited c.1895C>G (p.S632W) | GOF | LP | Not clear | generalized spike-wave on | Not clear | Not clear | (Li et al., 2018) |
| *HCN2* | F | Unknown | Unknown | Idiopathic photosensitive occipital epilepsy | Not clear | Idiopathic/genetic generalized epilepsy | None | Inherited c.1895C>G (p.S632W) | GOF | LP | Not clear | Unknown | Not clear | Not clear | (Li et al., 2018) |
| *HCN2* | M | FS | FS | Febrile seizures | Not clear | FS | None | Inherited c.1895C>G (p.S632W) | GOF | LP | Not clear | Unknown | Not clear | Not clear | (Li et al., 2018) |
| *HCN2* | 3y/M | Absence seizures | Generalized TCS | Absence seizures | Not clear | Idiopathic/genetic generalized epilepsy | None | Inherited c.1895C>G (p.S632W) | GOF | LP | Not clear | Generalized  SWDs | Not clear | Not clear | (Li et al., 2018) |
| *HCN2* | 6y/F | Photosensitive myoclonic seizures | Generalized TCS | Photo-sensitive genetic generalized epilepsy | Not clear | Idiopathic/genetic generalized epilepsy | Mild ID | Inherited c.736G>A (p.V246M) | GOF | LP | Not clear | Generalized  SWDs | Not clear | Not clear | (Li et al., 2018) |
| *HCN2* | M | Myoclonic with photosensitive generalized TCS | Myoclonic with photosensitive generalized TCS | Juvenile myoclonic epilepsy. | Not clear | Idiopathic/genetic generalized epilepsy | None | Inherited c.736G>A (p.V246M) | GOF | LP | Not clear | Not clear | Not clear | Not clear | (Li et al., 2018) |
| *HCN2* | M | Focal seizure | TCS | Generalized/Focal seizures/both | Not clear | Idiopathic/genetic generalized epilepsy | ADHD and abnormal behavior | Inherited c.736G>A (p.V246M) | GOF | LP | Not clear | Spikes and generalized SWDs, and | Not clear | Not clear | (Li et al., 2018) |
| **Names of the gene** | **Age at seizure onset/sex** | **Initial seizures** | **Seizure types during disease course** | **Syndrome/**  **phenotype** | **Presence of status epilepticus** | **Epilepsy syndromes re-classification** | **Other clinical features/ organs affected** | **Inheritance/nucleotide or protein change/location** | **Altered protein function** | **Silico modeling prediction/ACMG classification** | **MRI results** | **EEG finding** | **Therapies used** | **Prognosis** | **Reference** |
| *HCN2* | Not clear | FS | FS | FS | Not clear | FS | Not clear | Inherited heterozygous c. 377C.T (p.S126L ) | GOF | LP | Not clear | Unknown | Not clear | Not clear | (Nakamura et al., 2013) |
| *HCN2* | F | FS | FS | FS | Not clear | FS | Not clear | Inherited heterozygous c. 377C.T (p.S126L ) | GOF | LP | Not clear | Unknown | Not clear | Not clear | (Nakamura et al., 2013) |
| *HCN2* | Not clear | Not clear | Not clear | Idiopathic generalized epilepsy | Not clear | Idiopathic/genetic generalized epilepsy | Not clear | Inherited c.1580G>A (p.R527Q) | No changes in Ih current | The disease role is unclear | Not clear | Not clear | Not clear | Not clear | (Tang et al., 2008) |
| *HCN2* | Not clear | Not clear | Not clear | Genetic epilepsy with febrile seizures plus | Not clear | Idiopathic/genetic generalized epilepsy | Not clear | delPPP (p.719-721) | GOF | Not clear | Not clear | Not clear | Not clear | Not clear | (Dibbens et al., 2010) |
| *HCN2* | Not clear | Not clear | Not clear | Genetic epilepsy with febrile seizures plus | Not clear | Idiopathic/genetic generalized epilepsy | Not clear | delPPP (p.719-721) | GOF | Not clear | Not clear | Not clear | Not clear | Not clear | (Dibbens et al., 2010) |
| *HCN2* | Not clear | Not clear | Not clear | Genetic epilepsy with febrile seizures plus | Not clear | Idiopathic/genetic generalized epilepsy | Not clear | delPPP (p.719-721) | GOF | Not clear | Not clear | Not clear | Not clear | Not clear | (Dibbens et al., 2010) |
| *HCN2* | Not clear | Not clear | Not clear | Febrile seizures | Not clear | FS | Not clear | delPPP (p.719-721) | GOF | Not clear | Not clear | Not clear | Not clear | Not clear | (Dibbens et al., 2010) |
| **Names of the gene** | **Age at seizure onset/sex** | **Initial seizures** | **Seizure types during disease course** | **Syndrome/**  **phenotype** | **Presence of status epilepticus** | **Epilepsy syndromes re-classification** | **Other clinical features/ organs affected** | **Inheritance/nucleotide or protein change/location** | **Altered protein function** | **Silico modeling prediction/ACMG classification** | **MRI results** | **EEG finding** | **Therapies used** | **Prognosis** | **Reference** |
| *HCN2* | Not clear | Not clear | Not clear | Febrile seizures | Not clear | FS | Not clear | delPPP (p.719-721) | GOF | Not clear | Not clear | Not clear | Not clear | Not clear | (Dibbens et al., 2010) |
| *HCN2* | Not clear | Not clear | Not clear | Febrile seizures | Not clear | FS | Not clear | delPPP (p.719-721) | GOF | Not clear | Not clear | Not clear | Not clear | Not clear | (Dibbens et al., 2010) |
| *HCN3* | Mean age 40 y | Unknown | Unknown | Epilepsy plus SUDEP | Unknown | Not clear | Unknown | Unknown  K69R | Unknown | Nonsynonymous | Unknown | Unknown | Unknown | Died | (Tu et al., 2011) |
| *HCN3* | Mean age 40 y | Unknown | Unknown | Epilepsy plus SUDEP | Unknown | Not clear | Unknown | Unknown  P630L | Unknown | Nonsynonymous | Unknown | Unknown | Unknown | Died | (Tu et al., 2011) |
| *HCN4* | M | Generalized epilepsy | Generalized epilepsy | Familial Benign Myoclonic Epilepsy | Not clear | Idiopathic/genetic generalized epilepsy | Not clear | Familial heterozygous c.1648C > T (p.R550C) | LOF | LP | Not clear | Unknown | Not clear | Not clear | (DiFrancesco et al., 2019) |
| *HCN4* | M | Generalized epilepsy | Generalized epilepsy | Familial Benign Myoclonic Epilepsy | Not clear | Idiopathic/genetic generalized epilepsy | Not clear | Familial heterozygous c.1648C > T (p.R550C) | LOF | LP | Not clear | Unknown | Not clear | Not clear | (DiFrancesco et al., 2019) |
| **Names of the gene** | **Age at seizure onset/sex** | **Initial seizures** | **Seizure types during disease course** | **Syndrome/**  **phenotype** | **Presence of status epilepticus** | **Epilepsy syndromes re-classification** | **Other clinical features/ organs affected** | **Inheritance/nucleotide or protein change/location** | **Altered protein function** | **Silico modeling prediction/ACMG classification** | **MRI results** | **EEG finding** | **Therapies used** | **Prognosis** | **Reference** |
| *HCN4* | M/Infantile | LOC, myoclonus and dropping attacks | Generalized epilepsy | Familial Benign Myoclonic Epilepsy | Not clear | Idiopathic/genetic generalized epilepsy | Not clear | Familial heterozygous c.1648C > T (p.R550C) | LOF | LP | Not clear | Normal background, with generalized epileptiform activity prevalent in the anterior regions. | VPA | Seizure free | (Campostrini et al., 2018) |
| *HCN4* | M/Infantile | LOC, myoclonus and dropping attacks | Generalized epilepsy | Familial Benign Myoclonic Epilepsy | Not clear | Idiopathic/genetic generalized epilepsy | Not clear | Familial heterozygous c.1648C > T (p.R550C) | LOF | LP | Not clear | Normal background, with generalized epileptiform activity prevalent in the anterior regions | VPA | Seizure free | (Campostrini et al., 2018) |
| *HCN4* | Not clear | Generalized seizures | Generalized seizures | Genetic generalized epilepsy | Not clear | Idiopathic/genetic generalized epilepsy | Not clear | Inherited c.458A>G (p.E153G) | LOF | LP | Not clear | Not clear | Not clear | Not clear | (Becker et al., 2017) |
| *HCN4* | Mean age 40 y | Unknown | Unknown | Epilepsy plus SUDEP | Unknown | Not clear | Unknown | Unknown  Novel  G36E | Unknown | Nonsynonymous | Unknown | Unknown | Unknown | Died due to SUDEP | (Tu et al., 2011) |
| **Names of the gene** | **Age at seizure onset/sex** | **Initial seizures** | **Seizure types during disease course** | **Syndrome/**  **phenotype** | **Presence of status epilepticus** | **Epilepsy syndromes re-classification** | **Other clinical features/ organs affected** | **Inheritance/nucleotide or protein change/location** | **Altered protein function** | **Silico modeling prediction/ACMG classification** | **MRI results** | **EEG finding** | **Therapies used** | **Prognosis** | **Reference** |
| *HCN4* | Mean age 40 y | Unknown | Unknown | Epilepsy plus SUDEP | Unknown | Not clear | Unknown | Unknown  V759I | Unknown | Nonsynonymous | Unknown | Unknown | Unknown | Died due to SUDEP | (Tu et al., 2011) |
| *HCN4* | Mean age 40 y | Unknown | Unknown | Epilepsy plus SUDEP | Unknown | Not clear | Unknown | Unknown  Novel  G973R | Unknown | Nonsynonymous | Unknown | Unknown | Unknown | Died due to SUDEP | (Tu et al., 2011) |
| *HCN4* | Mean age 40 y | Unknown | Unknown | Epilepsy plus SUDEP | Unknown | Not clear | Unknown | Unknown  Novel  R1044W | Unknown | Nonsynonymous | Unknown | Unknown | Unknown | Died due to SUDEP | (Tu et al., 2011) |

**Abbreviations**: ADHD: Attention-deficit/hyperactivity disorder, CLB: clobazam, CLN: clonazepam, CS: clonic seizures, DD: developmental delay, EP: epilepsy, ETX: ethosuximide, EEG: electroencephalography, EIEE: early infantile epileptic encephalopathy, F: female, FS: febrile seizures, GSW: generalized spike waves, GOF: gain -of -function, ID: intellectual disability; LOC: loss of consciousness, LP: Likely pathogenic, LOF: loss-of-function, LEV: levetiracetam, LTG: lamotrigine, m= months, MRI: magnetic resonance imaging, MMPSI: malignant migrating partial seizures of infancy, M:male; PSW: polyspike waves, P=pathogenic, TCS: tonic-clonic seizures, TPM: topiramate, TS: tonic seizures, TCS: tonic-clonic seizure(s), STP: stiripentol, SUDEP: Sudden unexpected death in epilepsy, VPA: sodium valproate, y: years, ZNS: zonisamide.
