# Supplementary material for: The Contribution of HCN Channelopathies in Different Epileptic Syndromes, Mechanisms, Modulators, and Potential Treatment Targets: A Systematic Review
Source: Front Mol Neurosci. 2022 May 19;15:807202. doi: 10.3389/fnmol.2022.807202 (PMC9161305; doi:10.3389/fnmol.2022.807202)

**Data Sheet 1**

**A: Search strategies which were used.**

**PubMed**

1. HCN channel and epilepsy
2. HCN channel and seizures
3. HCN channel and convulsions

**Embase**

1. ('hcn channel'/exp OR 'hcn channel' OR (hcn AND channel)) AND ('epilepsy'/exp OR epilepsy OR 'seizures'/exp OR seizures OR 'convulsions'/exp OR convulsions)

**B: Prisma Flow Chart: Strategy of the literature search**


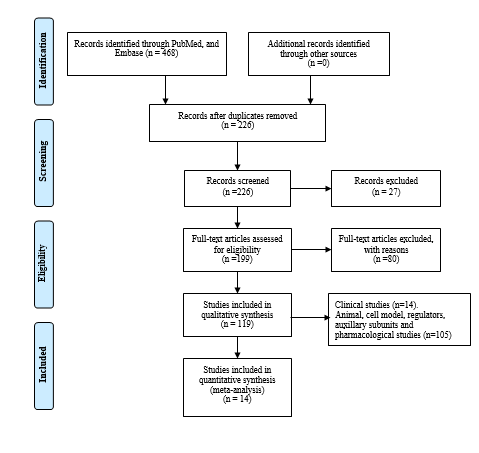

Supplement: Supplementary file 2 [file Data_Sheet_1.docx]
